# Distribution of oxidized DJ-1 in Parkinson’s disease-related sites in the brain and in the peripheral tissues: effects of aging and a neurotoxin

**DOI:** 10.1038/s41598-018-30561-z

**Published:** 2018-08-13

**Authors:** Yuichiro Mita, Yuto Kataoka, Yoshiro Saito, Takuma Kashi, Kojiro Hayashi, Asa Iwasaki, Takanori Imanishi, Tomohiro Miyasaka, Noriko Noguchi

**Affiliations:** 1 Systems Life Sciences Laboratory, Department of Medical Life Systems, Faculty of Life and Medical Sciences, Doshisha University, Kyoto, 610-0394 Japan; 2 Neuropathology, Department of Life and Medical Systems, Faculty of Life and Medical Sciences, Doshisha University, Kyoto, 610-0394 Japan

## Abstract

DJ-1 plays an important role in antioxidant defenses, and a reactive cysteine at position 106 (Cys106) of DJ-1, a critical residue of its biological function, is oxidized under oxidative stress. DJ-1 oxidation has been reported in patients with Parkinson’s disease (PD), but the relationship between DJ-1 oxidation and PD is still unclear. In the present study using specific antibody for Cys106-oxidized DJ-1 (oxDJ-1), we analyzed oxDJ-1 levels in the brain and peripheral tissues in young and aged mice and in a mouse model of PD induced using 1-methyl-4-phenyl-1,2,3,6-tetrahydropyridine (MPTP). OxDJ-1 levels in the brain, heart, and skeletal muscle were high compared with other tissues. In the brain, oxDJ-1 was detected in PD-related brain sites such as the substantia nigra (SN) of the midbrain, olfactory bulb (OB), and striatum. In aged wild-type mice, oxDJ-1 levels in the OB, striatum, and heart tended to decrease, while those in the skeletal muscle increased significantly. Expression of dopamine-metabolizing enzymes significantly increased in the SN and OB of aged DJ-1^−/−^ mice, accompanied by a complementary increase in glutathione peroxidase 1. MPTP treatment concordantly changed oxDJ-1 levels in PD-related brain sites and heart. These results indicate that the effects of physiological metabolism, aging, and neurotoxin change oxDJ-1 levels in PD-related brain sites, heart, and skeletal muscle where mitochondrial load is high, suggesting a substantial role of DJ-1 in antioxidant defenses and/or dopamine metabolism in these tissues.

## Introduction

*DJ-1*, also known as *PARK7*, is a causative gene of a familial form of Parkinson’s disease (PD)^1^. Its product DJ-1 plays an important role in antioxidant defenses^2^: the loss of DJ-1 function increases sensitivity to oxidative stress-induced cell death. DJ-1 belongs to the DJ-1/Hsp31/PfpI superfamily and functions as a covalent chaperone^3^. DJ-1 is a multifunctional protein that is involved in various physiological processes, including transcriptional regulation, mitochondrial function, and signal transduction^4^. DJ-1 regulates the function of Nrf2 and p53, and changes glutathione (GSH) metabolism and the expression levels of heat-shock proteins and uncoupling proteins^5^. DJ-1 regulates signal mediators of oxidative stress, such as PTEN and ASK1, via direct interactions with these proteins^6^. DJ-1 has also been identified as a regulator of the 20S proteasome^7^. The function of DJ-1 as a redox-activated chaperone could account for these multiple interacting proteins and functions.

DJ-1 possesses a reactive cysteine at position 106 (Cys106), which plays a critical role in the biological function of DJ-1^8^. Cys106 has a depressed p*K*a of 5.4 and its thiolate accepts a hydrogen bond from a protonated side chain of E18^9^. Cys106 is sequentially oxidized to cysteine sulfenic acid (Cys-SOH), cysteine sulfinic acid (Cys-SO_2_H), and cysteine sulfonic acid (Cys-SO_3_H)^10^. The acidic spot-shift of DJ-1 that is observed in 2D-polyacrylamide gel electrophoresis (PAGE) analysis arises from irreversible oxidation of this cysteine residue to either Cys-SO_2_H or Cys-SO_3_H. The former is chemically unstable and easily oxidized to the latter under normoxic conditions. However, Cys-SO_2_H at position 106 is stable because of the surrounding amino acid residues, and the Cys-SO_2_H form is postulated to be the active form of DJ-1. Previous studies have shown that mutation of Glu18 to Asn (E18N), which enhanced Cys106 oxidation and stabilized the Cys-SO_2_H form, has a protective effect against mitochondrial damage, while the E18D mutation diminished both Cys106 oxidation and cytoprotective effects^11,12^. Oxidation of Cys106 to Cys-SO_3_H leads to loss of biological function. Additionally, 2D-PAGE analysis of human brain samples showed the presence of more acidic form of DJ-1, which has been confirmed not only by western blotting but also by mass spectrometry^13^; however, the precise modification of the more acidic form of DJ-1 has not been elucidated. It is thought that DJ-1 acts as an oxidative stress sensor, detecting cellular redox status through the oxidation of Cys106 and altering the activity of signal mediators and the expression levels of genes involved in antioxidant defenses^8,14^.

PD is a progressive, age-related, neurodegenerative disorder, characterized by bradykinesia, rigidity, and tremors. These symptoms are caused by the degradation of dopamine neurons in the pars compacta of the substantia nigra (SN) in the midbrain and the subsequent depletion of striatal dopamine^15^. Tyrosine hydroxylase (TH) catalyzes the first synthesis step of catecholamines such as dopamine and adrenaline, and functions as the rate-limiting enzyme. Dopaminergic neurons in the SN express TH^16^. SN dopaminergic neurons are rich in reactive oxygen species (ROS) because the enzymatic and nonenzymatic metabolism of dopamine leads to the generation of ROS, including superoxide anions, hydrogen peroxide (H_2_O_2_), and hydroxyl radicals^17^. Monoamine oxidase (MAO), which is bound to the outer membrane of the mitochondria, catalyzes the oxidation of monoamines such as dopamine, and enzymatically generates H_2_O_2_. Oxidative stress is a crucial mediator in the pathogenesis of PD. Increased levels of oxidation products, of lipids, proteins, and nucleic acids have been demonstrated in SN cells of PD patients^17,18^. An increase in the amounts of oxidants such as copper and iron and a decrease in the amounts of antioxidants such as GSH and GSH peroxidase 4 (GPx4) have been reported in the SN of PD patients^19^.

Brain sites other than the SN and peripheral tissues are also impaired in PD patients, particularly during the presymptomatic phase^20^. For example, degeneration of the olfactory bulb (OB) and concomitant disorders of olfaction have been reported, and a smell test has been tried as an early indicator of disease onset^21^. In addition, post-mortem cardiac samples show decreased levels of axons expressing TH, which acts as a marker for sympathetic axons in peripheral tissues, indicating degeneration of the cardiac nerves in PD patients^22^. Meta-iodobenzylguanidine (MIBG), an analogue of noradrenaline, is actively taken up and stored in sympathetic nerves, and cardiac uptake of radiolabeled MIBG has been used for the diagnosis of PD^23^.

The identification of biochemical markers of PD has received much attention. Biomarkers related to oxidative stress, such as oxidized proteins, are considered to be candidates because of the involvement of oxidative stress in PD. From this point of view, Cys106-oxidized DJ-1 (oxDJ-1) could be an interesting candidate^24^. To detect oxDJ-1 conveniently, we prepared specific antibodies (Abs) against oxDJ-1 to allow oxDJ-1-specific western blotting, immunostaining, and enzyme-linked immunosorbent assay (ELISA) to be carried out^13,25^. Since Cys-SO_2_H is chemically unstable, it is still unclear whether our oxDJ-1-specific Abs recognized the Cys106-SO_2_H and/or Cys106-SO_3_H forms of DJ-1; several types of data, including the immunoreactivity against recombinant oxDJ-1 and peptides containing Cys106-SO_3_H, suggest that our Abs recognized both forms of oxDJ-1^13,25^. Immunohistochemical analysis suggests that, in the SN of the midbrain, oxDJ-1 levels increase in the early phases of PD and then decrease at later stages of PD in patients with dementia who have already lost almost all of their dopaminergic neurons^13^. Previous analysis also suggests that unmedicated PD patients (those who have been diagnosed with PD but not yet started on medications) have increased erythrocyte oxDJ-1 levels compared with PD patients who have been treated with L-DOPA and/or dopamine agonists, and with healthy subjects^25^. Thus, the evidence suggests that DJ-1 oxidation occurs in the erythrocytes and brain of PD patients, particularly during the early disease phases. An association between oxDJ-1 levels in erythrocytes and cardiac uptake of MIBG has also been suggested^26^. An elevation of oxDJ-1 levels in blood has been observed in mouse and nonhuman primate models of PD induced by the administration of neurotoxins such as 1-methyl-4-phenyl-1,2,3,6-tetrahydropyridine (MPTP) and 6-hydroxydopamine (6-OHDA)^25,27^. The evidence suggests that DJ-1 oxidation in blood occurs in both PD patients and in animal models of PD. However, the molecular link between DJ-1 oxidation in dopaminergic-neuronal cells and peripheral tissues is not understood.

Several studies using DJ-1^−/−^ (DJ-1 KO) animal models have reported diverse physiological roles of DJ-1, not only in the nervous system but also in whole-body metabolism, and have demonstrated that the aging process affects the disorder seen in these biological systems^5,28^. An aging-dependent disorder of motor function in DJ-1 KO mice has been reported, and a possible role of DJ-1 in the regulation of microtubule dynamics, which is important in axon guidance and outgrowth of neurites, has been identified^5,29^. Hypersensitivity of DJ-1 KO mice to MPTP and oxidative stress has also been reported^5,30^. On the other hand, studies using isolated brain mitochondria suggest increased respiration-dependent H_2_O_2_ consumption in DJ-1 KO mice^31^, and several reports have showed that dopamine neurons in the SN of DJ-1 KO mice retain the normal number and have normal levels of dopamine^5,32^. Species-specific transcriptional regulation of the TH promoter by DJ-1 has been demonstrated^33^. However, the relationship between the phenotype of DJ-1 KO mice and DJ-1 function is still unclear.

In the present study, we used oxDJ-1-specific Ab to investigate the distribution of oxDJ-1 in mouse brain and peripheral tissues, and analyzed the effects of aging and MPTP administration on oxDJ-1 levels. The alterations in the antioxidant defenses and dopamine metabolism in oxDJ-1-rich brain sites of DJ-1 KO mice were also investigated.

## Results

### Distribution of oxDJ-1 in mouse brain and peripheral tissues

**Figure 1:**
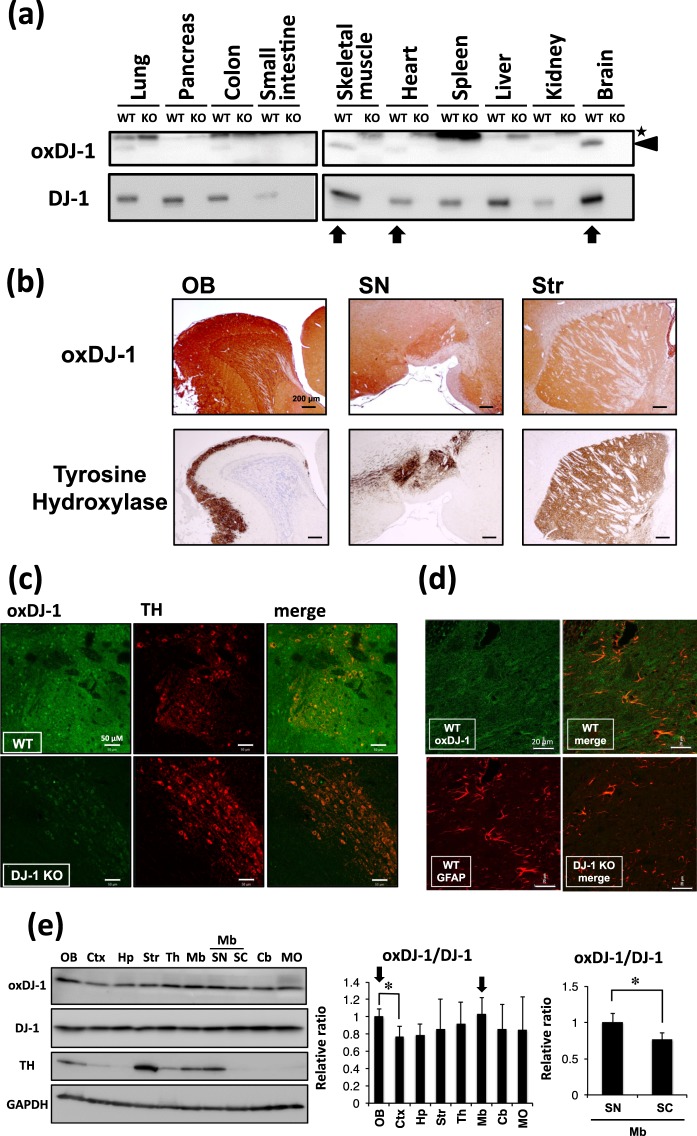
Distribution of oxidized DJ-1 in mouse brain and peripheral tissues. **(a)** Lysates from each tissue of young wild-type (WT) and DJ-1^−/−^ mice were subjected to western blotting using anti-oxDJ-1 and anti-DJ-1 Abs. The specific oxDJ-1 band is indicated by a black arrowhead and a nonspecific band is indicated by an asterisk. **(b)** Comparison of oxDJ-1 immunoreactivity with TH immunostaining in the olfactory bulb (OB), substantia nigra (SN), and striatum (Str). Sagittal sections from wild-type C57BL/6J mice were stained with anti-oxDJ-1 Ab and anti-TH Ab. Oxidized DJ-1 IR was observed around TH-positive neurons. **(c,d)** Confocal images of oxDJ-1 in SN dopaminergic neurons and astrocytes. A section containing the SN was immunostained with anti-oxDJ-1 Ab and anti-TH Ab **(c)** or anti-glial fibrillary acidic protein (GFAP) Ab **(d)** and then visualized using fluorescence confocal microscopy. A scale bar is shown in each figure. **(e)** Western blot analyses of oxDJ-1 in separated brain tissues. Protein lysates of each brain site were subjected to western blot analyses using Abs against DJ-1, oxDJ-1, TH, and glyceraldehyde-3-phosphate dehydrogenase (GAPDH). The relative band densities of oxDJ-1 normalized to DJ-1 were calculated and are presented as mean ± SD (n = 6). *P < 0.05, Tukey-Kramer test, ANOVA (middle panel) and Student’s *t*-test (right panel). Ctx, cortex; Hp, hippocampus; Th, thalamus; Mb, midbrain; SC, superior colliculus; Cb, cerebellum; MO, medulla oblongata.

The distribution of oxDJ-1 in each tissue was determined by western blotting using anti-oxDJ-1 monoclonal Ab (mAb). The specificity of each band was confirmed by its absence in biological samples from DJ-1 KO mice. The highest oxDJ-1 protein levels were detected in whole brain, followed by skeletal muscle and heart (Fig. 1a). In addition, a faint oxDJ-1 band was detected in lung and colon (Fig. 1a). Western blot analysis of DJ-1 suggested that DJ-1 protein levels were high in brain and skeletal muscle, and the next highest were in liver, lung, and pancreas (Fig. 1a). DJ-1 protein levels in the small intestine and kidney were relatively low.

The distribution of oxDJ-1 in mouse brain was investigated by immunohistochemistry using oxDJ-1-specific mAb, and compared with that of TH. The specificity of oxDJ-1 immunostaining was confirmed using brain sections from DJ-1 KO mice (Supplementary Figs S1 and S2). We observed intense oxDJ-1 immunoreactivity around sites staining for TH, such as the SN, striatum (Str), and OB (Fig. 1b). In the SN area of the midbrain, oxDJ-1 immunoreactivity was diffuse and broader, and was also indicated in the TH-positive neurons and astrocytes (Supplementary Fig. S3). OxDJ-1 immunoreactivity was confirmed in the SN dopaminergic neurons using confocal microscopy (Fig. 1c), but this was absent in the SN of DJ-1 KO mice. Astrocyte expression of oxDJ-1 in the SN and its absence in DJ-1 KO mice was also confirmed using double immunostaining with anti-glial fibrillary acidic protein (GFAP) (Fig. 1d). These results suggest that in the SN, oxDJ-1 was present in TH-positive neurons and astrocytes, and also in other types of cells.

To analyze quantitatively the distribution of oxDJ-1 protein in brain, extracted mouse brain was separated into eight areas as shown in Supplementary Fig. S4. Protein lysates were prepared from each area and subjected to western blotting. Separation of the catecholaminergic neuron-rich areas was confirmed by the distribution of TH: high levels of TH were observed in the OB, Str, and midbrain (Fig. 1e and Supplementary Fig. S5). Midbrain was further separated into SN and superior colliculus (Supplementary Fig. S4), and the high TH levels in SN were confirmed (Fig. 1e and Supplementary Fig. S5). In the western blot analysis of these separated brains, oxDJ-1 and DJ-1 were detected in all regions, and DJ-1 levels were approximately the same in these separated brain samples (Fig. 1e). We found that oxDJ-1 levels differed slightly in the brain regions and that the ratio of oxDJ-1 to DJ-1 tended to be high in the TH-rich sites such as the OB and midbrain, and particularly in the SN (Fig. 1e).

### Distribution of oxDJ-1 in each brain tissue and effects of aging

**Figure 2:**
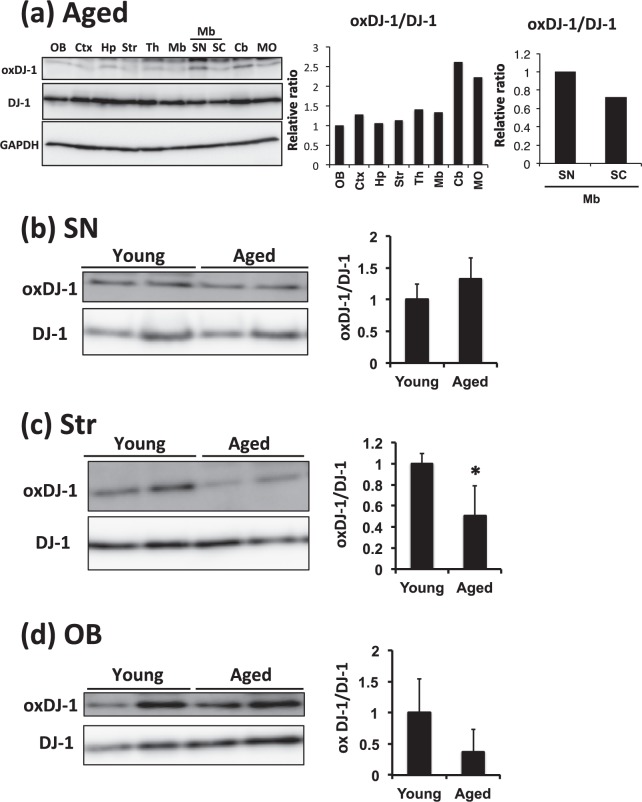
The effects of aging on oxDJ-1 levels in the brain. **(a)** Western blot analyses of oxDJ-1 in separated brain tissues of aged mice. Protein lysates of each brain site were subjected to western blot analyses. The relative band densities of oxDJ-1 normalized to DJ-1 were calculated and are presented as mean (n = 2). Abbreviations are same with Fig. 1. (**b**–**d**) Protein lysates of the substantia nigra (SN, **b**), striatum (Str, **c**), and olfactory bulb (OB, **d**) of young (9 weeks) and aged (130 weeks) WT mice were separated by SDS-PAGE and then subjected to western blotting using each Ab. The densities of each band were determined and the relative densities of oxDJ-1 relative to DJ-1 were calculated and are presented as mean ± SD (n = 3). *P < 0.05, Student’s *t*-test.

To explore the effects of aging on DJ-1 oxidation, the distribution of oxDJ-1 and DJ-1 in separated brain tissues of aged mice was determined, although the sample number was limited (n = 2). The oxDJ-1 bands were slight but detectable in all separated brain regions, and DJ-1 levels were approximately similar in these samples (Fig. 2a). In a comparison with the distribution of oxDJ-1 in each brain region of young mice (Fig. 1e), we found that the oxDJ-1:DJ-1 ratio of aged mice tended to be slightly low in the OB and Str, while oxDJ-1 levels tended to be high in the cerebellum (Fig. 2a). The levels of oxDJ-1 in the SN showed the trend to be high compared with the superior colliculus of midbrain, as observed in the young mice (Fig. 2a). We further compared the levels of oxDJ-1 in multiple samples of separated brain tissues from young (9 weeks of age) and aged (130 weeks of age) mice. For the dopaminergic neuron-rich brain sites, no change in oxDJ-1 levels was observed in the SN, but oxDJ-1 levels in the Str and OB tended to decrease in aged mice (Fig. 2b–d). Modification of DJ-1 in the Str and the OB was further evaluated by 2D-PAGE combined with western blotting using anti-DJ-1 mAb. The results of 2D-PAGE analysis showed several spots indicating native and oxidized DJ-1, and in addition, an acidic form of DJ-1 with an unknown modification (UmDJ-1) was detected (Supplementary Figs S6 and S7). The intensity of each spot was determined, and compared between young and aged mice. However, obvious differences between each spot were not observed (Supplementary Figs S6 and S7).

### Distribution of oxDJ-1 in each peripheral tissue and effects of aging

**Figure 3:**
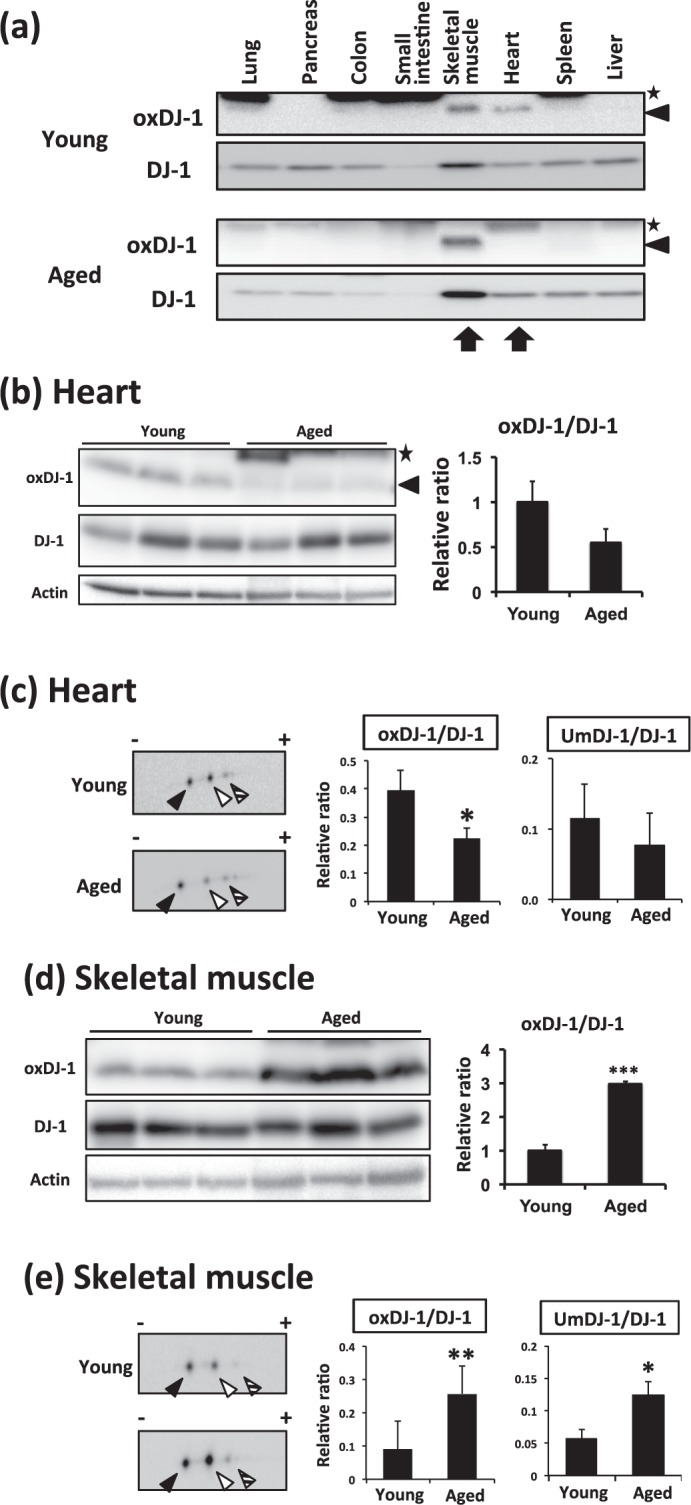
Change of oxDJ-1 levels in the heart and skeletal muscle of aged mice. **(a)** Lysates from each tissue of young (9 weeks) and aged (130 weeks) wild-type (WT) mice were subjected to western blotting using anti-oxDJ-1 and anti-DJ-1 Abs. The change in oxDJ-1 levels in the skeletal muscle and heart is shown. **(b–e)** Proteins in lysates from hearts **(b** and **c)** and skeletal muscle **(d** and **e)** of young and aged WT mice were separated by SDS-PAGE **(b** and **d)** or 2D-PAGE **(c** and **e)** and then subjected to western blotting using the indicated Ab. The densities of each band were determined and the relative densities of oxDJ-1 compared with DJ-1 were calculated and are presented as mean ± SD (b, n = 3). In 2D-PAGE, black, white, and striped arrowheads indicate native DJ-1, oxDJ-1, and unknown modified DJ-1 (UmDJ-1). The densities of each spot were determined and the relative densities of oxDJ-1 and UmDJ-1 relative to DJ-1 were calculated and are presented as mean ± SD (C, n = 3). *P < 0.05, Student’s *t*-test.

We next compared the oxDJ-1 levels in peripheral tissues of young and aged mice. As shown in Fig. 3a, we found a trend to decreased oxDJ-1 levels in aged heart. The comparison using multiple heart samples from young and aged mice suggested that oxDJ-1 levels in the hearts of aged mice tended to decrease (Fig. 3b). In 2D-PAGE analysis, a comparison of spots from young and aged mice showed weak intensity for oxDJ-1, and oxDJ-1 levels in heart tissue of aged mice were significantly lower than those in young mice (Fig. 3c). The UmDJ-1 levels also tended to decrease in aged mice. Collectively, these results suggest that oxDJ-1 levels decrease in the heart tissue of aged mice.

Comparison of the oxDJ-1 levels in peripheral tissues also suggests an increase in DJ-1 oxidation in the skeletal muscle of aged mice (Fig. 3a). Western blotting of multiple samples showed a remarkable elevation of oxDJ-1 levels in the skeletal muscle of aged mice (Fig. 3d), and 2D-PAGE analysis showed an intense oxDJ-1 spot in aged skeletal muscle (Fig. 3e). The specificity of the anti-oxDJ-1 mAb was confirmed in 2D-PAGE analysis of skeletal muscle (Supplementary Fig. S8). The intensities of each spot were compared, and we found that oxDJ-1 levels in the skeletal muscle of aged mice were significantly higher than those in young mice (Fig. 3e). In addition, a significant increase in UmDJ-1 levels was observed in aged mice (Fig. 3e). Collectively, these results suggest the elevation of oxDJ-1 in skeletal muscle of aged mice.

### The increase of glutathione peroxidase 1 in DJ-1 KO mice and effects of aging

**Figure 4:**
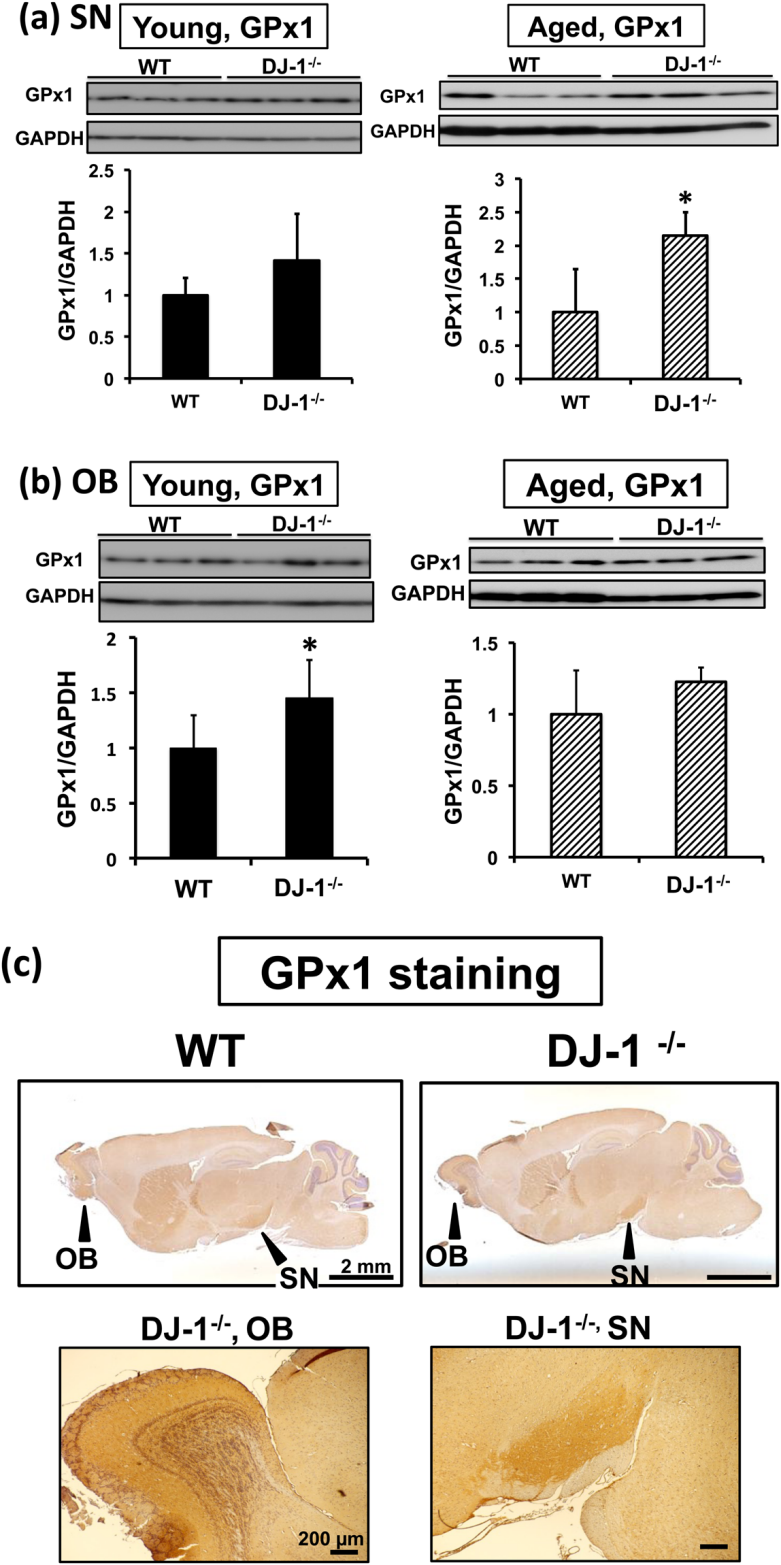
Elevation of glutathione peroxidase 1 (GPx1) levels in Parkinson’s disease-related brain sites of DJ-1^−/−^ (DJ-1 KO) mice. **(a,b)** Young (9 weeks of age, left panel) and aged (more than 100 weeks of age, right panel) mice were analyzed. Protein lysates of the substantia nigra (SN, a) and olfactory bulb (OB, b) of wild-type (WT) and DJ-1 KO mice were subjected to western blot analyses using anti-GPx1 Ab. The relative band densities of GPx1 per GAPDH were calculated and presented as mean ± SD (n = 3–5). *P < 0.05, Student’s *t*-test. **(c)** Immunohistochemical distribution of the GPx1 protein in the mouse brain. Sagittal sections from young WT and DJ-1 KO mice brains were stained with anti-GPx1 Ab. GPx1 immunoreactivity was present throughout the brain from both mice, and was dense in the OB and SN of mouse brain. A scale bar is shown in each figure part.

Oxidized DJ-1 in the PD-related brain areas in mice suggested an important role of DJ-1 in antioxidant defenses in these sites. We next investigated the alteration of antioxidant defenses in young DJ-1 KO mice (9 weeks of age). Because of the importance of DJ-1 in the defense against hydroperoxides, the levels of the major antioxidant enzymes involved in removal of hydroperoxides, selenoenzyme GPxs, were evaluated by western blotting. We found that GPx1, but not GPx4, tended to increase in the SN of young DJ-1 KO mice (Fig. 4a and Supplementary Fig. S9), and found that GPx1 was significantly elevated in the OB of young DJ-1 KO mice (Fig. 4b). It has been reported that in DJ-1 KO mice, the aging process chronically increases oxidative stress and induces several disorders of motor function and metabolism^32,34^. Therefore, we also evaluated the effects of aging on GPx levels in these sites using tissues from aged mice (more than 100 weeks of age). We found that GPx1 was significantly increased in the SN of aged DJ-1 KO mice (Fig. 4a). An age-dependent increase of GPx1 in the SN of DJ-1 KO mice was also confirmed (Supplementary Fig. S10). The levels of GPx1 in the OB and of GPx4 in the SN of aged DJ-1 KO mice also tended to increase (Fig. 4b and Supplementary Fig. S9). Immunohistochemical analysis suggested a dense distribution of GPx1 in the SN and OB (Fig. 4c). We next determined the levels of the reduced form of GSH in the SN and OB of these mice. There was no significant difference between the SN of young and aged wild-type (WT) and DJ-1 KO mice (Supplementary Fig. S11), while in the OB of aged mice, we found that GSH levels were significantly lower in DJ-1 KO mice than in WT mice (Supplementary Fig. S12), suggesting a decrease in antioxidant defenses and an increase in oxidative stress in the OB of aged DJ-1 KO mice. Taken together, these results suggest that there is a compensatory increase of GPx1 in the SN and OB of DJ-1 KO mice and confirm the importance of DJ-1 in antioxidant defenses at these sites.

### The effects of DJ-1 KO and aging on dopaminergic neurons

**Figure 5:**
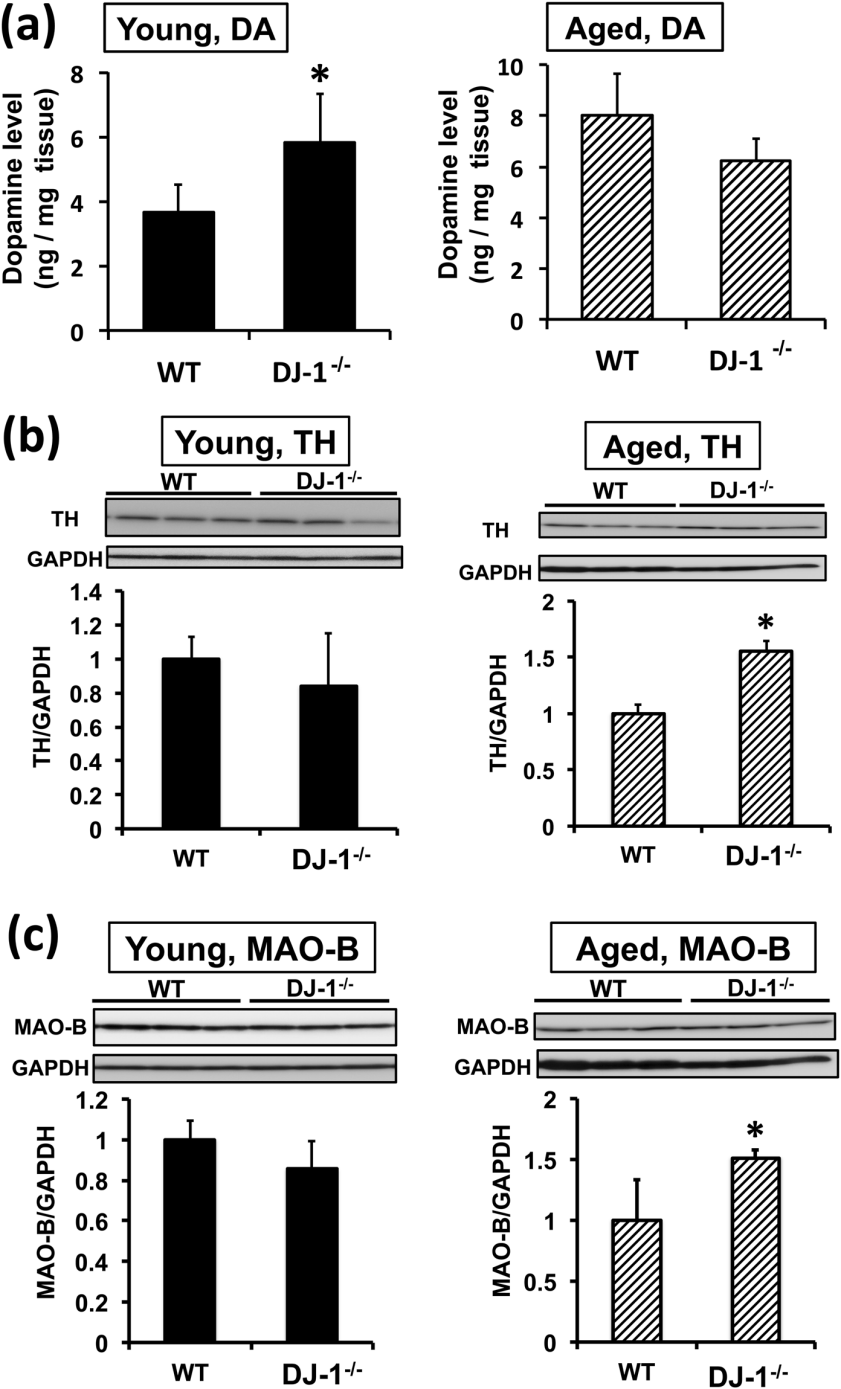
The alteration of dopamine metabolism in DJ-1^−/−^ (DJ-1 KO) and aged mice. **(a–c)** Striatal levels of dopamine **(a)** and the substantia nigra levels of tyrosine hydroxylase (TH, **b**) and monoamine oxidase-B (MAO-B, **c**) in wild-type (WT) and DJ-1 KO mice were determined in young (9 weeks of age, left panel) and aged (more than 100 weeks of age, right panel) mice (n = 4–5). The relative band densities of TH or MAO-B relative to GAPDH were calculated. *P < 0.05, Student’s *t*-test.

We next determined the change in dopamine metabolism between young and aged mice. In young DJ-1 KO mice, dopamine levels in the Str increased significantly, suggesting that DJ-1 suppresses dopamine levels (Fig. 5a), but TH levels in the SN, a rate-limiting enzyme in the synthesis of dopamine, were unchanged (Fig. 5b). However, in aged DJ-1 KO mice, dopamine levels in the Str were not significantly altered, while TH levels in the SN were significantly increased (Fig. 5a,b). The levels of MAO type B (MAO-B), a mitochondrial enzyme required for the oxidative degradation of dopamine, were also determined by western blotting. We found a significant increase of MAO-B levels in the SN of aged DJ-1 KO mice (Fig. 5c). Determining the levels of TH and MAO-B in the Str is important; however, in the present study, we could not determine these levels because of limited amounts of Str samples. On the other hand, we could determine the levels of TH and MAO-B in the OB. We found that MAO-B levels, but not TH levels, in the OB of aged DJ-1 KO mice increased significantly (Supplementary Figs S13 and S14). Collectively, these results suggest the acceleration of dopamine metabolism in aged DJ-1 KO mice.

### Alteration in oxDJ-1 levels induced by MPTP treatment

**Figure 6:**
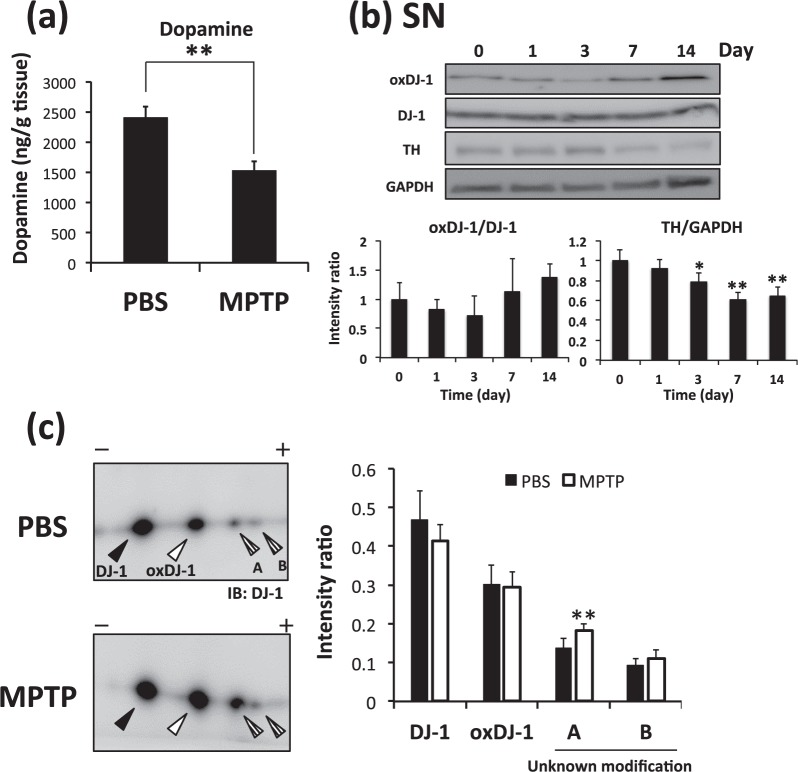
The change in DJ-1 oxidation in the substantia nigra (SN) of MPTP-treated mice. After the administration of PBS and MPTP (15 mg/kg, 3 times i.p.), each brain was separated and subjected to analysis. **(a)** Striatal levels of dopamine in mice were determined 3 days after MPTP administration (n = 5). **P < 0.01, Student’s *t*-test. **(b)** Protein lysates of the SN of MPTP-treated mice were subjected to western blot analyses using each specific Ab. The relative band densities of oxDJ-1 relative to DJ-1 and tyrosine hydroxylase (TH) relative to GAPDH were calculated and are presented as mean ± SD (n = 4). **P < 0.01, *P < 0.05, Tukey-Kramer test, ANOVA, when compared with day 0. **(c)** Modification of DJ-1 in the SN of MPTP-treated mice was evaluated using 2D-PAGE and western blotting for DJ-1. The filled triangles and open triangles indicate native and oxidized DJ-1, respectively. The striped triangles A and B indicate DJ-1 with an unknown modification. The relative densities of each spot were estimated. Graphs display the relative densities of each spot compared with PBS control as mean ± SD (n = 5). **P < 0.01, Student’s *t-*test.

We further investigated the change in oxDJ-1 levels in the separated brain of MPTP-treated mice using western blotting. A significant decrease of dopamine levels in the Str by 3 days after the administration of MPTP was confirmed (Fig. 6a). The oxDJ-1 levels in the SN also tended to decrease at 3 days after MPTP treatment, and then to increase at 14 days; however, these changes were not significant (Fig. 6b). TH levels in the SN were significantly decreased from day 3 (Fig. 6b). The oxDJ-1 levels in the OB showed a similar trend to those in the SN (Supplementary Fig. S15). Modification of DJ-1 in the SN at day 3 was further evaluated by 2D-PAGE combined with western blotting using anti-DJ-1 mAb. The intensity of each spot was determined, and compared between treated and nontreated mice. We observed the generation of UmDJ-1 in the SN of MPTP-treated mice (Fig. 6c).

**Figure 7:**
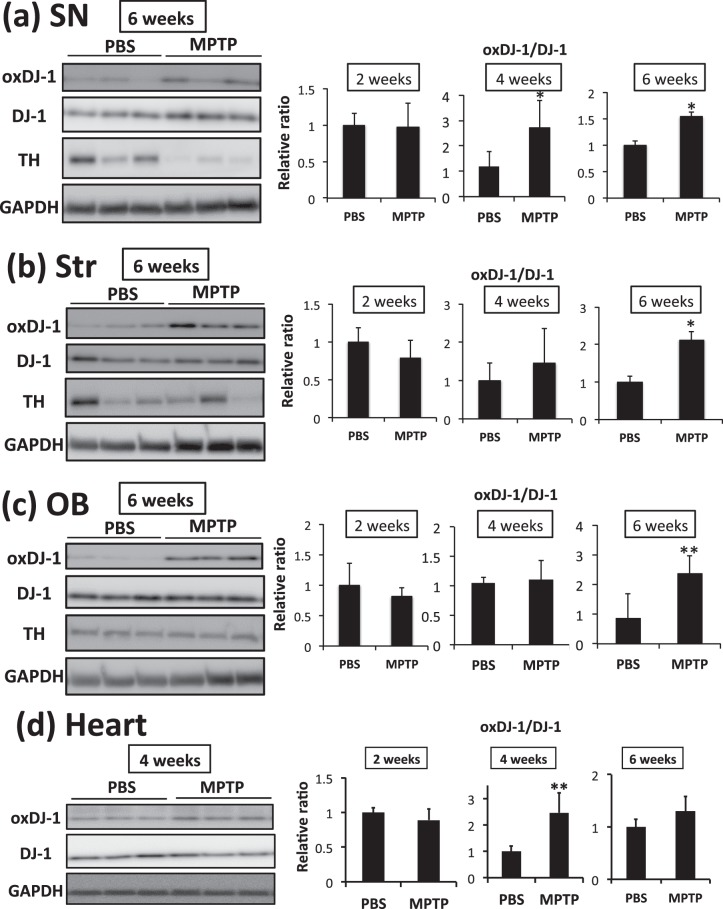
Elevation of oxidized DJ-1 in the brain and heart of MPTP-treated mice. After the administration of PBS and MPTP (15 mg/kg, 3 times i.p.), each brain and heart were separated and subjected to analysis. **(a–d)** Protein lysates of the substantia nigra (SN, **a**), striatum (Str, **b**), olfactory bulb (OB, **c**), and heart (**d**) of MPTP-treated mice were subjected to western blot analyses using each specific Ab. The relative band densities of oxDJ-1 relative to DJ-1 were calculated and are presented as mean ± SD (n = 6). **P < 0.01, *P < 0.05, Student’s *t*-test.

Because previous studies suggested that oxDJ-1 was elevated in blood and brain several weeks after MPTP treatment, we further determined oxDJ-1 levels in each brain area up to 6 weeks after treatment. A significant increase of oxDJ-1 levels was observed in the SN from 4 weeks after MPTP treatment, and continued until 6 weeks after treatment (Fig. 7a). Similarly, oxDJ-1 levels were significantly increased in the Str and OB 6 weeks after treatment (Fig. 7b,c). Decreased TH levels in the SN were observed 6 weeks after MPTP treatment (Fig. 7a), whereas TH levels in the Str and OB did not obviously decrease (Fig. 7b,c). On the other hand, oxDJ-1 levels in the Ctx, cerebellum (Cb), and hippocampus (Hp) did not change significantly (Supplementary Figs S16–S18). It has been reported that MPTP modulates heart noradrenalin^35^, and we have previously observed an association between oxDJ-1 levels in blood and cardiac uptake of MIBG^26^. Therefore, we evaluated DJ-1 oxidation in heart tissue, and found that it increased significantly at 4 weeks after MPTP treatment and tended to increase after 6 weeks after treatment (Fig. 7d). In the case of skeletal muscle, a significant change in oxDJ-1 levels was not observed (Supplementary Fig. S19). These results suggest that DJ-1 oxidation proceeds in the PD-related brain sites and in heart tissue from 4 weeks after MPTP treatment.

## Discussion

Previous studies have shown that oxidation of Cys106 of DJ-1 proceedes under oxidative stress and tends to change during the course of PD^8,14^. In the present study, we used oxDJ-1-specific Ab to detect oxDJ-1 in the brain and peripheral tissues, and found that physiological metabolism, aging, and neurotoxin change oxDJ-1 levels in PD-related brain sites, heart, and skeletal muscle where mitochondrial load is high. This suggests a significant role of DJ-1 in antioxidant defenses and/or mitochondrial function in these tissues. This study further emphasizes the biological significance of DJ-1 oxidation and the tight relationship between DJ-1 oxidation, mitochondrial function, and the pathophysiology of PD.

Analysis of oxDJ-1 levels in the brain and peripheral tissues suggests that the brain is rich in oxDJ-1, followed by skeletal muscle and heart (Fig. 1a). It has been known that these sites have high mitochondrial load with both mitochondrial mass and respiratory demand. The substantial connection between oxDJ-1 and mitochondrial function has been demonstrated^4,36^, and it has been shown that Cys-SO_2_H-driven mitochondrial localization is important for the neuroprotective role of DJ-1^12,37^. Thus, this evidence further supports the significant relationship between DJ-1 oxidation and mitochondrial function, implying the potential role of mitochondrial metabolism in DJ-1 oxidation.

Immunohistochemical and western blot analyses of mouse brain suggested the presence of oxDJ-1 in the SN of midbrain, the OB, and the Str, where TH is abundantly expressed (Fig. 1). It is known that dopamine metabolism generates ROS in an enzymatic and nonenzymatic manner, suggesting that DJ-1 is oxidized in PD-related brain sites. Western blot analysis of samples from aged mice suggested that oxDJ-1 levels in the OB and the Str tended to decrease in aged C57BL/6J mice and that oxDJ-1 levels in the heart were also decreased in aged mice (Figs 2 and 3). Previous biophysical studies of oxDJ-1 show that Cys106-SO_2_H stabilizes DJ-1, while Cys106-SO_3_H destabilizes DJ-1^8,11^. Our oxDJ-1-specific Abs might recognize both the Cys106-SO_2_H and Cys106-SO_3_H forms of DJ-1, and this point is considered as a limitation of the present study. It is therefore unclear whether the decrease of oxDJ-1 in aged mice is due to the extensive degradation of Cys106-SO_3_H and/or the decrease of DJ-1 oxidation to form Cys106-SO_2_H/SO_3_H. A decrease in antioxidant responses and beneficial effects of antioxidants in the brain and heart of aged mice have been reported^38,39^. It appears that the decrease in oxDJ-1 levels might be related to the decay of antioxidant function in the brain and heart of aged mice.

A trend toward increasing levels of GPx1, a major cellular reductant of H_2_O_2_^40^, in the SN and OB of DJ-1 KO mice suggest a significant role of DJ-1 in antioxidant defenses in these brain sites. In the SN of aged DJ-1^−/−^ mice, a compensatory increase of GPx1 was observed (Fig. 4a), while in the SN of aged WT mice, oxDJ-1 was detected at the same level as in young WT mice (Fig. 2b), suggesting that DJ-1 plays a significant antioxidative role in the SN, particularly in aged mice. On the other hand, in the OB of DJ-1^−/−^ mice, a compensatory increase of GPx1 was observed in young DJ-1^−/−^ mice (Fig. 4b), and GSH levels in the OB of aged DJ-1^−/−^ mice decreased significantly (Supplementary Fig. S10), suggesting the increase of oxidative stress in the OB of aged DJ-1^−/−^ mice. It is notable that in the OB of aged WT mice, oxDJ-1 levels tended to decrease (Fig. 2f). DJ-1 is thought to play an essential antioxidative role in the OB. Collectively, it is considered that GSH-GPx1 axis in the OB and the SN of DJ-1 KO mice is sensitively changing, and it appears that the change might occur earlier in the OB than in the SN. In the future, smell testing of DJ-1 KO mice might be interesting.

Increasing evidences suggest that DJ-1 plays a role in regulating cellular dopamine levels, and species-specific transcriptional regulation of the *TH* promoter by DJ-1 has been reported^33,41–43^. DJ-1 acts as a coactivator of polypyrimidine tract-binding protein-associated splicing factor (PSF), which binds to the promoter region of the *TH* gene to repress its expression. Human DJ-1 binds to PSF to sequester the PSF-corepressor complex, whereas mouse DJ-1 cannot restore PSF^33^. This species-specific difference suggests the lack of a decrease in dopamine levels in DJ-1 KO out mice^33^. In aged mice, whereas dopamine levels in the Str were similar in WT and KO mice, levels of TH and MAO-B in the SN of DJ-1 KO mice increased significantly. It is important to determine TH and MAO-B levels in the Str to understand the change of dopamine metabolism; however, in the present study, these levels were not determined because of a lack of Str protein samples. Since our results suggest that metabolism of dopamine might be active, we then tried to determine the change in dopamine metabolite levels such as those of 3,4-dihydroxyphenylacetic acid. However, the determination of these metabolites was impossible because of the low quantity of samples from aged mice. We suggest that an acceleration of dopamine metabolism could result in the generation of ROS, which in turn could lead to increased GPx1 in the SN of aged KO mice; this would compensate for the loss of DJ-1 and inhibit oxidative stress.

MPTP treatment resulted in changes in oxDJ-1 levels in brain sites rich in TH-positive neurons (Figs 6 and 7). MPTP is metabolized by MAO in glia cells and is converted to the neurotoxic compound MPP^+^, which is incorporated via the dopamine transporter^44^. This toxic mechanism explains the selective effects of MPTP on brain sites containing dopaminergic neurons. At day 3 after MPTP injection, the trend to decreasing oxDJ-1 levels in the SN and the significant increase in the more acidic UmDJ-1 suggested severe injury to this area of the brain. After this acute response, oxDJ-1 levels were significantly increased in the SN at 4 and 6 weeks after MPTP treatment, suggesting a chronic response to the injury of dopaminergic neurons. An increase in blood oxDJ-1 levels in the chronic phase has been observed in MPTP-treated mice (significant difference after 4 weeks)^27^ and in a nonhuman primate model (significant difference after 10 weeks)^25^. Interestingly, the specific effect on particular brain sites, i.e., the significant increase of oxDJ-1 levels in the SN, Str, and OB, but not in the Ctx, Cb, and Hp, was observed during this chronic response. Rojo *et al*. have reported the levels of MPTP and MPP^+^ in the SN, Str, and OB of MPTP-treated mice (20 mg/kg, one time, ip), and the rapid increase of MPP^+^ levels in the SN and Str compared with those in the OB^45^. That study has also demonstrated the longer accumulation of MPP^+^ in the SN and OB, but not in the Str, and the obvious decrease of TH levels in the SN and Str, but not in the OB, 30 days after MPTP injection. In that study, the lower sensitivity of the OB to MPTP toxicity compared with that of the SN and Str has been discussed, which might be caused by the low expression of dopamine transporter. In the present study, we found that in the chronic phase, TH levels in the SN, but not in the Str and OB, obviously decreased (Fig. 7), suggesting the implication of a different level of sensitivity to MPTP. OxDJ-1 levels in heart, but not in the skeletal muscle, also increased significantly during the chronic phase (Fig. 7). It has been reported that MPTP treatment depletes norepinephrine and dopamine, but not TH levels to a great extent, in mouse heart^35,46^. It appears that a disorder of catecholaminergic neurons in heart tissues might result in DJ-1 oxidation during the chronic phase. Taken together, these results suggest that oxDJ-1 levels elevate coordinately in PD-related brain sites and in heart. At present, it is unclear why oxDJ-1 levels in these tissues increase at the chronic phase. Previous studies have reported the significant increase of striatal levels of dopamine from 1 week after MPTP treatment, suggesting that the repair of tissues might proceed several weeks after MPTP injection. Recent evidence suggested a role for mitochondrial metabolism in tissue repair^47^. In addition, the beneficial role of NADPH oxidase (NOX) in tissue regeneration and wound healing has been reported, and the role of ROS as a signaling molecule in tissue repair has been discussed^48^. It might be considered that mitochondrial and/or NOX-derived ROS related to tissue repair induce DJ-1 oxidation, and further studies are necessary to resolve the biological meaning of the chronic increase of oxDJ-1 levels in PD models.

In conclusion, this study clearly shows that oxDJ-1 is present in PD-related brain sites and peripheral tissues such as heart and skeletal muscle, which have high mitochondrial load, suggesting the role of physiological and mitochondrial metabolism in the DJ-1 oxidation. We found that oxDJ-1 levels were changed by aging and PD-related stimuli, not only in the brain but also in the heart, with coordinated alterations in DJ-1 oxidation in these sites, suggesting a substantial role of DJ-1 in antioxidant defenses in these tissues. Our observations might help us to understand the pathophysiological alterations in PD, particularly the relation of mitochondria, and to develop efficient biomarkers of this neurodegenerative disease, which will lead to the development of antioxidant therapy for PD.

## Materials and Methods

### Materials

Rabbit anti-DJ-1 mAb (clone EP2816Y) was purchased from GeneTex, Irvine, CA; mouse anti-β actin mAb (AC-15) was obtained from Sigma-Aldrich, St. Louis, MO; rabbit anti-TH polyclonal Ab (pAb, AB152) was purchased from Merck Millipore, Billerica, MA; rabbit anti-GPx1 pAb (ab22604), rabbit anti-GPx4 mAb (clone EPNCIR144, ab125066), rabbit anti-MAO type B (MAO-B) mAb (clone EPR7103, ab125010), and mouse anti-glyceraldehyde-3-phosphate dehydrogenase (GAPDH) mAb (clone 6C5, ab8245) were obtained from Abcam, Cambridge, MA; mAb against oxDJ-1 [clone M106 and clone M149 (MABN1773, Merck Millipore)] were prepared as previously described^13^. Other chemicals used were of the highest quality commercially available.

### Animals

All animal experiments described in this study fully confirmed to the guidelines outlined in the Guide for the Care and Use of Laboratory Animals of Japan and were approved by the Animal Care Committee of the Doshisha University (approval no. A17020). C57BL/6 J mice were obtained from Shimizu Laboratory Supplies (Kyoto, Japan). DJ-1 KO mice (B6.Cg-Park7^tm1Shn^/J) were purchased from the Jackson Laboratory, Bar Harbor, ME. Mice were housed under a 12 h light/dark cycle and allowed free access to food and water.

### Immunohistochemistry

Paraffin-embedded sections of WT and DJ-1 KO mice were prepared as previously described^13^. Whole brains of WT and DJ-1 KO mice were removed immediately after anesthetization, and subsequent transcardial perfusion was performed with PBS and 4% paraformaldehyde in PBS. The samples were then fixed overnight with paraformaldehyde in PBS at 4 °C and embedded in paraffin.

Paraffin-embedded sections (5 µm) from each brain tissue were stained using immunohistochemistry with anti-oxDJ-1 mAb (clones M149) or anti-DJ-1 mAb (clones EP2816Y). Briefly, after deparaffinization, the sections were incubated with 10% normal goat serum in 50 mmol/L Tris-HCl (pH 7.4) containing 150 mmol/L NaCl (TBS) at room temperature, and then with primary Ab (2 µg/mL). Then, the sections were incubated with the biotinylated secondary Ab, the avidin-biotin-peroxidase complex (Vector Laboratories, Burlingame, CA), and 3,3-diaminobenzidine in the presence of H_2_O_2_. The sections were then lightly counterstained with hematoxylin. All stained sections were photographed under a light microscope connected to a charge-coupled device camera.

For confocal microscopy, bound Abs were visualized with Alexa 488Y conjugated anti-mouse IgG or Alexa 568Y conjugated anti-rabbit IgG (Molecular Probes, Eugene, OR). Specimens were observed using a laser-scanning microscope (LSM 710 ConfoCor 3; Carl Zeiss, Thornwood, NY) equipped with Zeiss Efficient Navigation 2009 software.

### Dissection of the brain

Dissection of the brain was conducted as described previously^49^. Briefly, the whole brain was removed rapidly and placed on an ice-cold glass plate with the ventral surface facing upward. Each brain site was separated from the whole brain. Tissue samples were frozen in liquid nitrogen and stored at −80 °C.

### SDS-PAGE and 2D-PAGE for western blotting

Tissue samples were taken for western blotting after perfusion with saline and immediately were frozen by liquid nitrogen. Tissue samples stored at −80 °C were homogenized in lysis buffer (50 mM Tris-HCl pH 7.5, 150 mM NaCl, 1% NP40, 0.1% SDS, 1% sodium deoxycholic acid with a cocktail of protease inhibitor [Nacalai Tesque, Kyoto, Japan] and phosphatase inhibitor [PhosSTOP, Roche, Mannheim, Germany]). The homogenates were spun for 5 min at 15,000 × g for 5 min, and the supernatant was used for western blot analysis. The protein concentration was determined by using a bicinchoninic acid protein assay kit (Pierce Biotechnology, Rockford, IL, USA) with BSA as the standard.

For western blot analysis, each tissue sample was reduced and denatured in 63 mmol/L Tris-HCl (pH 6.8) containing 1% mercaptoethanol, 2% SDS, 5% sucrose, and 0.012% bromophenol blue for 5 min at 95 °C. The reduced protein mixture was then separated on a 12.5% SDS-PAGE gel. For the first dimension of 2D-PAGE, immobilized pH gradient gel strips (pH 4–7; non-linear, 7 and 13 cm, GE Healthcare Bio-Science, Uppsala, Sweden) were used. The samples were mixed with sample buffer [9 M urea, 5% 3-[(3-cholamidopropyl)dimethylammonio]propanesulfonate, 65 mM dithioerythritol (DTE), and 0.5% ampholyte (pH 4–7)], applied on a gel, and rehydrated for 18 h. The electrophoresis voltage was increased stepwise to either 5,000 or 8,000 V at a maximum current of 200 mA for 3–5 h. Each strip was equilibrated in 50 mM Tris-HCl (pH 8.8) containing 6 M urea, 2% SDS, 30% glycerol, and 20 mM DTE for 20 min, and then separated on a 12.5% SDS-PAGE gel.

After separation by either SDS-PAGE or 2D-PAGE, the samples were transferred to an Immobilon-P Transfer Membrane (Millipore, Bedford, MA) for western blot analysis. The membranes were blocked in 5% skimmed milk powder (Snow Brand Milk Products, Tokyo, Japan) dissolved in Tris-buffered saline (pH 7.4) containing 0.1% Tween 20 (TBS-T), and incubated with anti-DJ-1 mAb or anti-oxDJ-1 mAb (clone M106, 1 μg/ml) at 4 °C for 18 h, washed with TBS-T, incubated with HRP-conjugated secondary Abs for at least 1 h, and washed with TBS-T. The immunoreactivity was visualized with Immobilon Western (Millipore) and an LAS-4000 luminescence imager (Fujifilm, Tokyo, Japan). The relative densities were determined with the MultiGauge software (Fujifilm). The full blot corresponding to the main figures are shown in the Supplementary Figures as follows: Supplementary Figs S20, 1a,e; Supplementary Figs S21, 2a–d; Supplementary Figs S22, 3a,b,d. Nonspecific bands with high molecular weight were observed in an oxDJ-1 blot using mouse anti-oxDJ-1 mAb (M106 clone), but not in a DJ-1 blot using rabbit anti-DJ-1 mAb (EP2816Y clone). Major nonspecific bands at approximately 80 and 25 kDa were detected by secondary Ab (anti-mouse IgG-HRP), and we suggest that these nonspecific bands were mainly caused by the reactivity of secondary Ab against mouse proteins in tissues samples.

### Determination of dopamine contents

Dopamine levels in the striatum were measured as described previously^49^. Briefly, extracted striatum samples were homogenized in 0.2 M perchloric acid containing 100 µM ethylenediaminetetraacetate. After centrifugation, the pH of the supernatant was adjusted to 3 by adding 1 M sodium acetate. The samples were subjected to high-performance liquid chromatography with an electrochemical detector (HTEC-500, Eicom, Kyoto, Japan). The results were shown as nanogram of dopamine per g of tissue.

### Animal experiments

All animal experiments described in this study fully conformed to the guidelines outlined in the Guide for the Care and Use of Laboratory Animals of Japan and were approved by the Animal Care Committee of Doshisha University (approval no. A16064). We did not randomize animals, and we performed all experiments without blinding to the investigator. Statistical methods were not used to determine sample size. MPTP (15 mg/kg, i.p.) was administered three times to 8-week-old male C57BL/6J mice at 2-h intervals. The controls were administered saline at similar intervals.

### Statistical analysis

The difference between determinations was statistically analyed with either Student’s *t*-test or analysis of variance (ANOVA) using Tukey-Kramer test for multiple comparisons. Values of *P* < 0.05 were considered as significant.

## Electronic supplementary material

Supplementary Figure

## Data Availability

All relevant data are available from the authors upon reasonable request.
