# Supplementary material for: Distribution of oxidized DJ-1 in Parkinson’s disease-related sites in the brain and in the peripheral tissues: effects of aging and a neurotoxin
Source: Sci Rep. 2018 Aug 13;8:12056. doi: 10.1038/s41598-018-30561-z (PMC6089991; doi:10.1038/s41598-018-30561-z)
Supplement: Supplementary file 1 — Supplementary Figure [file 41598_2018_30561_MOESM1_ESM.pdf]

**Y Mita et al.,**
**Supplementary Figure**

**Title: Distribution of oxidized DJ-1 in Parkinson's disease-related sites in the brain and in the peripheral tissues: effects of aging and a neurotoxin**

Authors: Yuichiro Mita,[1‡] Yuto Kataoka,[1‡] Yoshiro Saito,[1*] Takuma Kashi,[1] Kojiro Hayashi,[1] Asa Iwasaki,[1] Takanori Imanishi,[1] Tomohiro Miyasaka,[2] and Noriko Noguchi[1*]

[1]Systems Life Sciences laboratory, [2]Neuropathology, Department of Life and Medical Systems, Faculty of Life and Medical Sciences, Doshisha University, Kyoto 610-0394, Japan.

*Correspondence should be addressed to Yoshiro Saito and Noriko Noguchi, Systems Life Sciences laboratory, Department of Life and Medical Systems, Faculty of Life and Medical Sciences, Doshisha University, 1-3 Miyakodani, Tatara, Kyotanabe, Kyoto 610-0394, Japan. E-mail: ysaito@mail.doshisha.ac.jp and nnoguchi@mail.doshisha.ac.jp

# S1

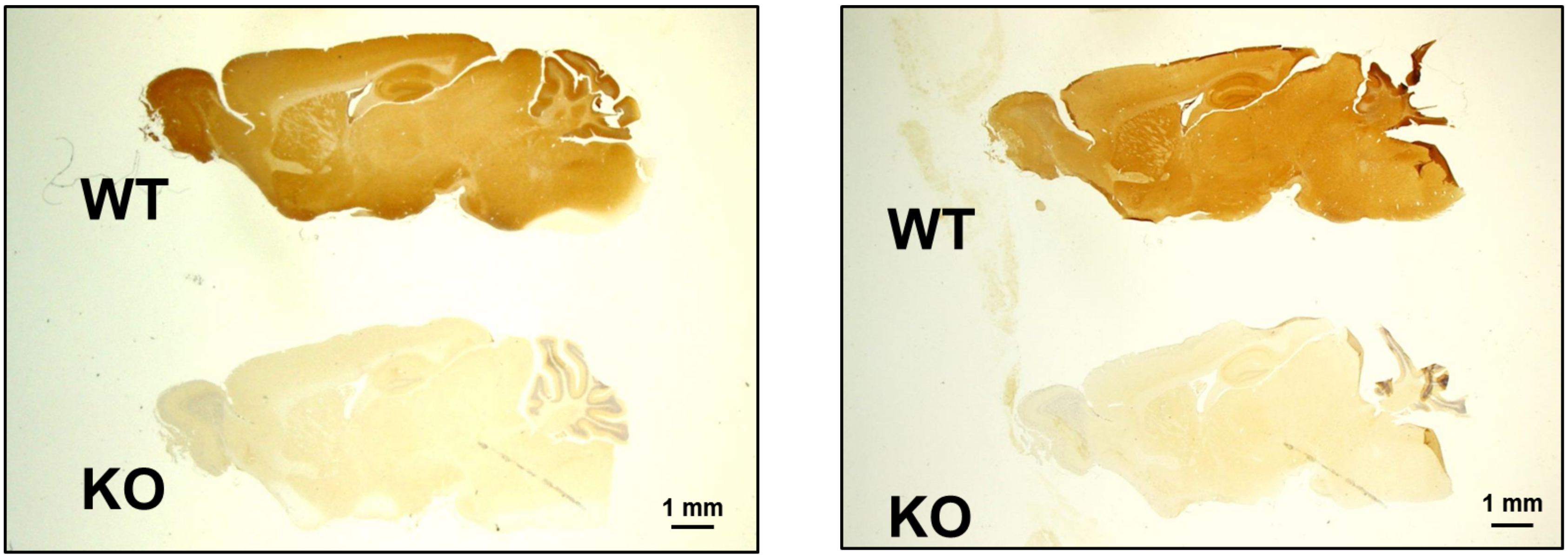


oxDJ-1 stain

DJ-1 stain

**Supplementary Figure S1 | Immunohistochemical analysis of oxDJ-1 and DJ-1 protein in the mouse brain.** Sagittal sections from wild-type (WT) and DJ-1$^{-/-}$ (DJ-1 KO) mice brains were stained with anti-oxDJ-1 and anti-DJ-1 Ab. The disappearance of immunoreactivity of each Ab was confirmed in the DJ-1 KO mouse brain. A scale bar is shown in each figure.

# S2

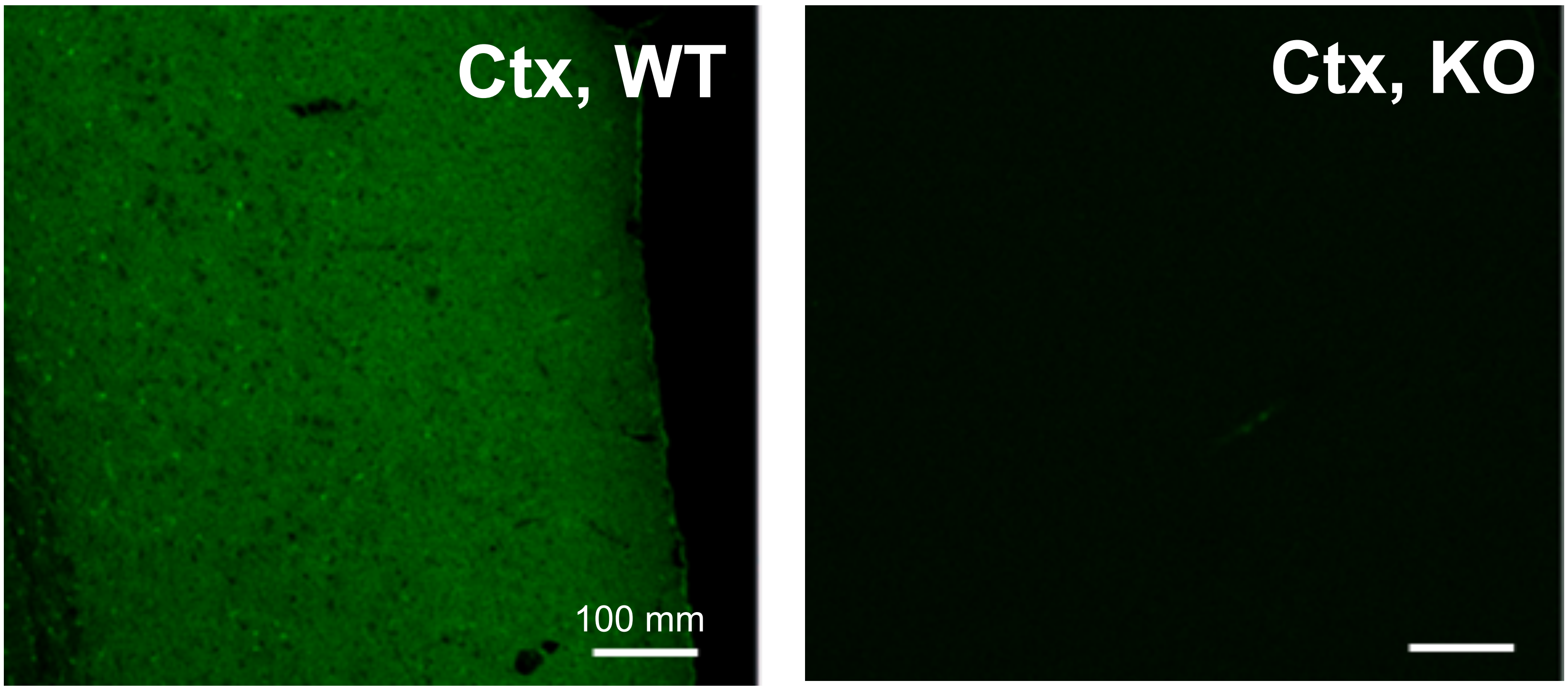


oxDJ-1 stain

**Supplementary Figure S2 | Immunohistochemical analysis of oxDJ-1 protein in the mouse brain.** Sagittal sections of the cortex (Ctx) of wild-type (WT) and DJ-1$^{-/-}$ (DJ-1 KO) mice brains were stained with anti-oxDJ-1 Ab and then visualized using fluorescence confocal microscopy. The disappearance of oxDJ-1 immunoreactivity was confirmed in the DJ-1 KO mouse brain. A scale bar is shown in each figure.

Supplementary Fig. S1-2, Y. Mita, et al.

# S3

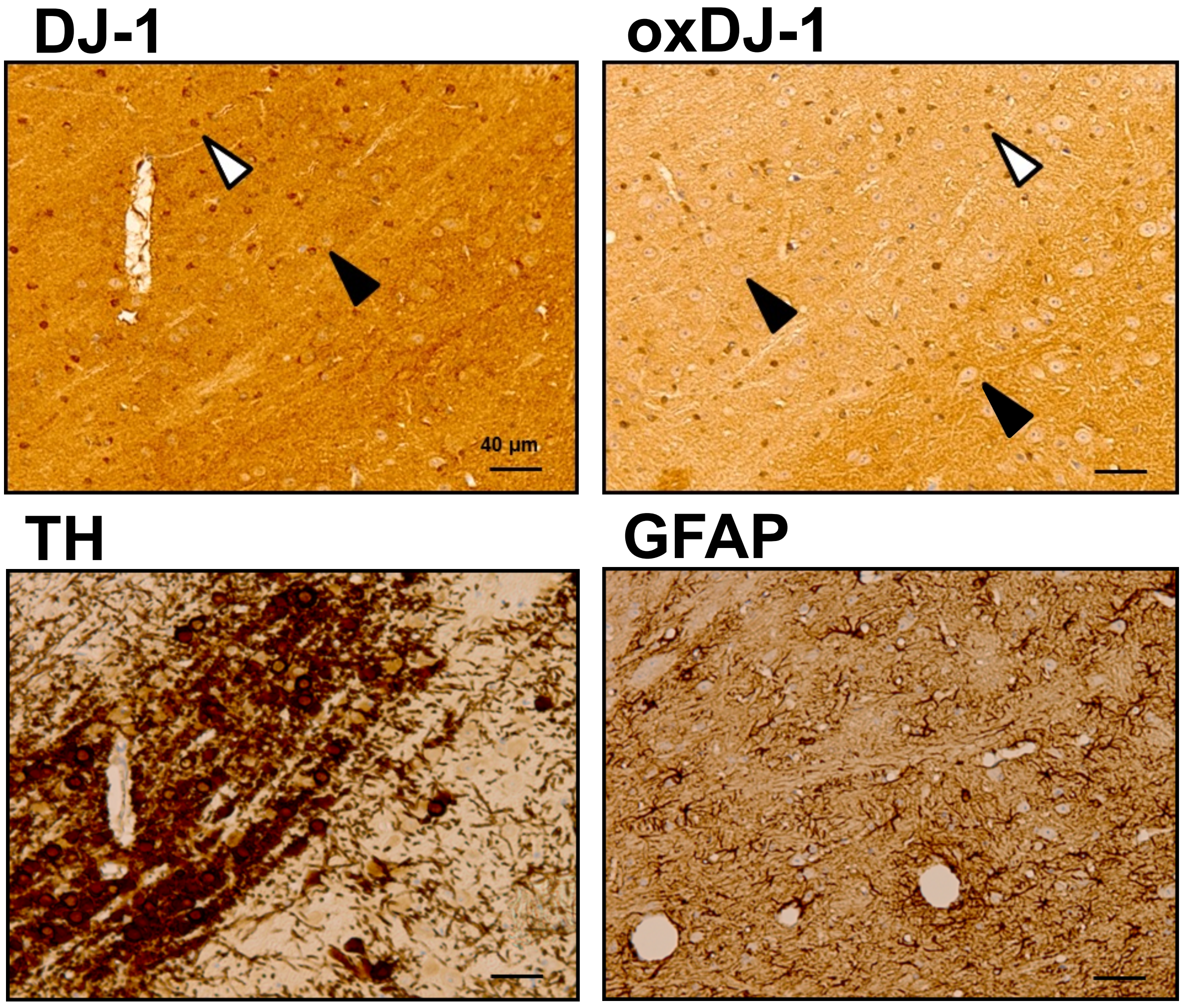


**Supplementary Figure S3 | Staining results at higher magnification of the substantia nigra.** Sagittal sections of the substantia nigra (SN) of wild-type mice brains were stained with indicated Ab. DJ-1 and oxDJ-1 immunoreactivity was enriched in the perikaryon of neuronal cells (indicated by black arrowhead) and in the nuclei of glial cells (indicated by white arrowhead). Serial sections were also stained by anti-tyrosine hydroxylase (TH) and anti-glial fibrillary acidic protein (GFAP). A scale bar is shown in each figure.

# S4

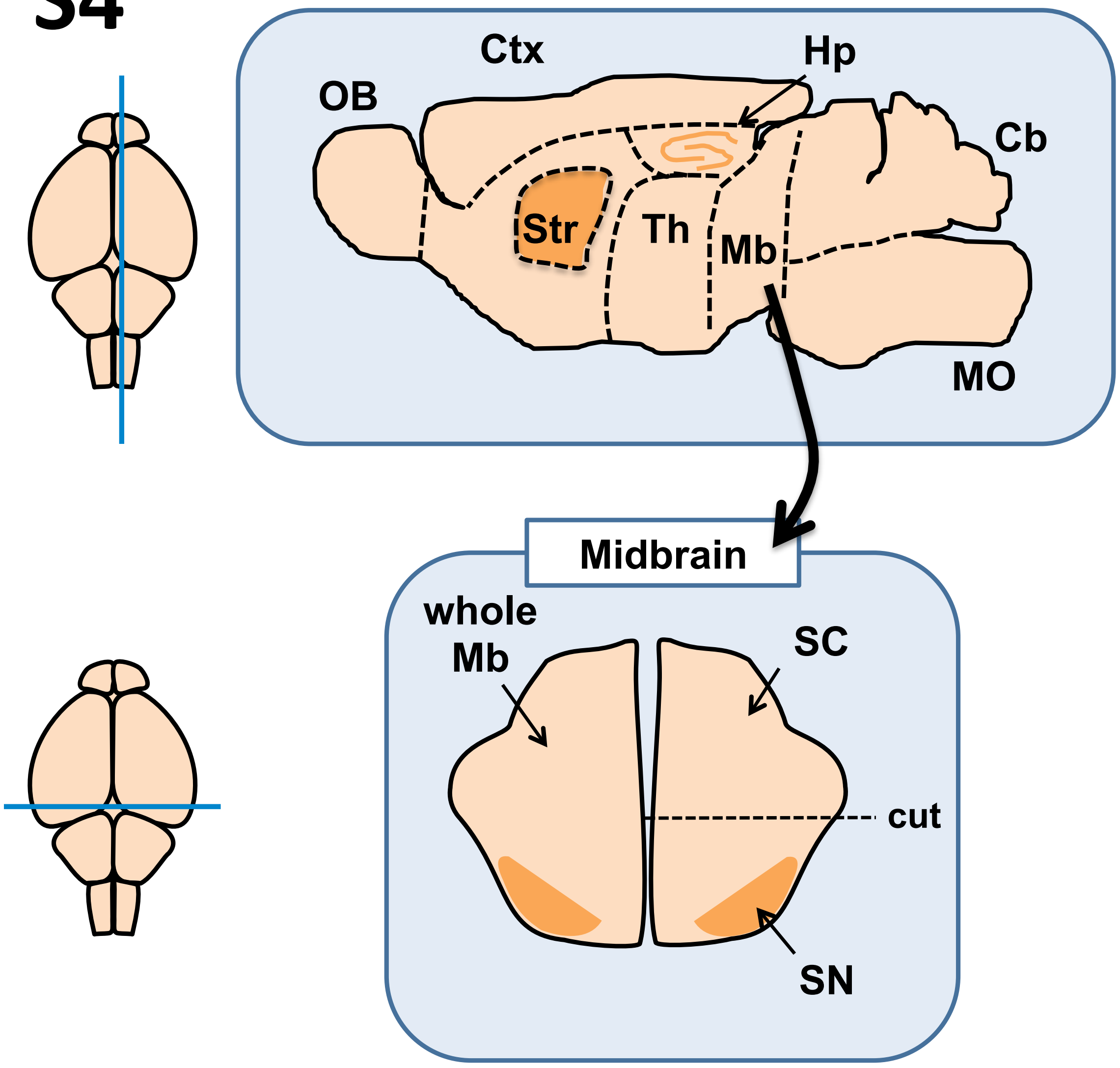


**Supplementary Figure S4 | Pattern diagram of separation of the mouse brain.** Extracted mouse brain was cut and separated to 8 sites as shown in upper panel. Midbrain (Mb) was further cut and separated to the superior colliculus (SC) and substantia nigra (SN) as shown in lower panel. Separated brain sites were used for further analysis.

**Supplementary Fig. S3-4, Y. Mita, et al.**

# S5

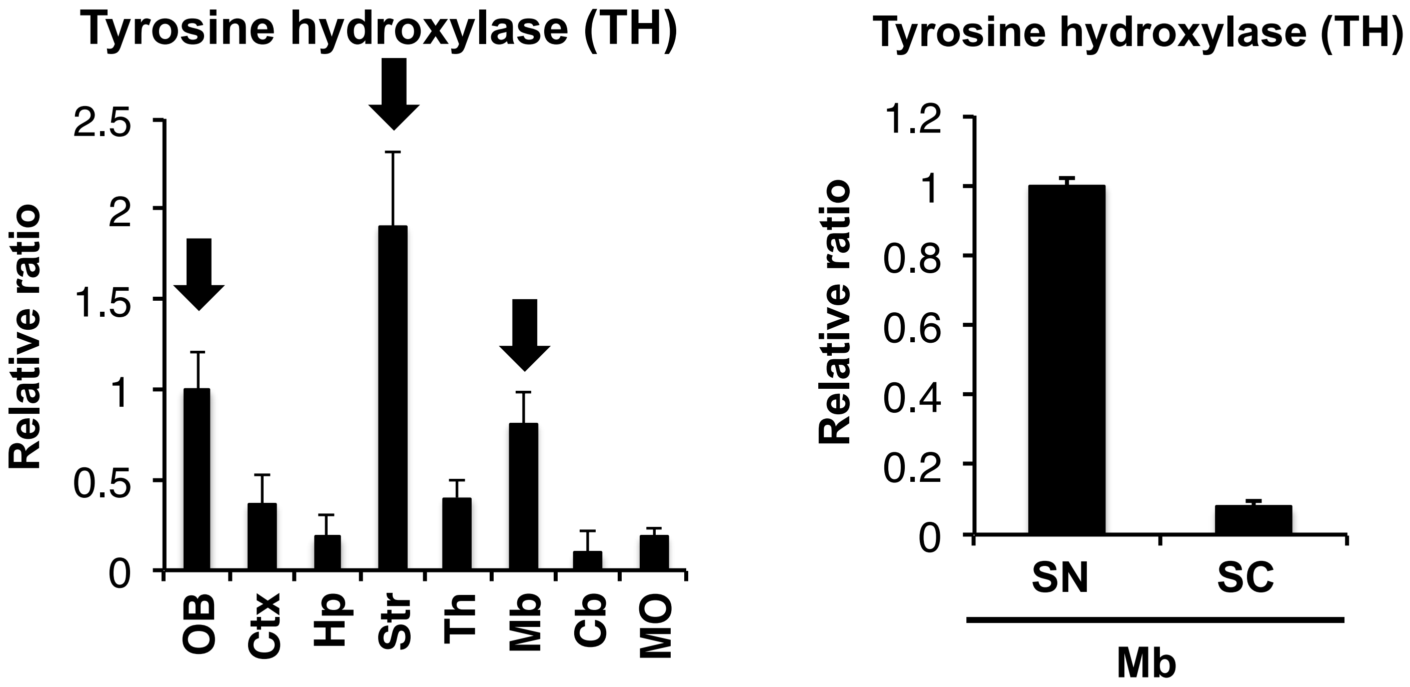


**Supplementary Figure S5 | Western blot analyses of tyrosine hydroxylase (TH) in separated brain tissue.** Protein lysates of each brain site was subjected to western blot analyses using Abs against TH and glyceraldehyde-3-phosphate dehydrogenase (GAPDH). The relative band densities of TH normalized to GAPDH were calculated and are presented as mean ± SD (n = 6). Abbreviations were same with Fig. 1D.

# S6: Str

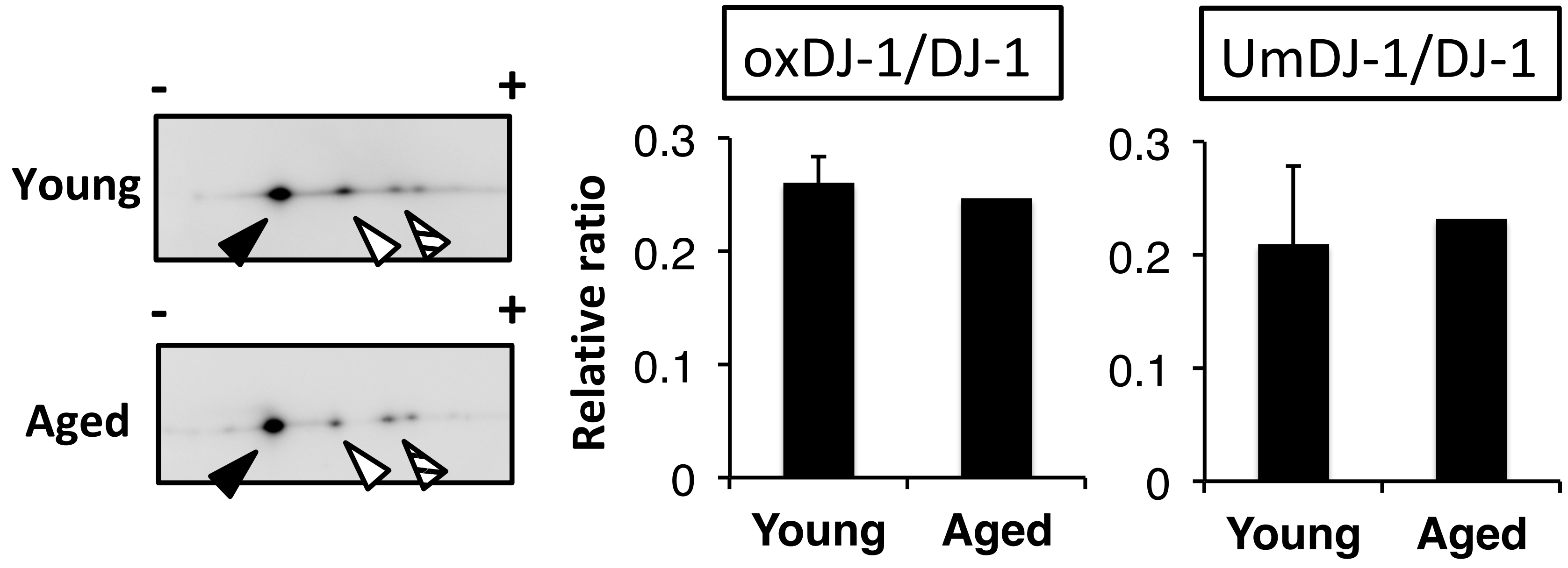


**Supplementary Figure S6 | 2D-PAGE analysis of DJ-1 in the striatum of aged mice.** Proteins in lysates from the striatum of young (n = 3) and aged (n = 2) mice were separated by 2D-PAGE and then subjected to western blotting using anti-DJ-1 and anti-oxDJ-1 Ab. Black, white, and striped arrowheads indicate native DJ-1, oxDJ-1, and unknown modified DJ-1 (UmDJ-1). A spot corresponding to oxDJ-1 was only observed in 2D western blot analysis using anti-oxDJ-1 Ab.

# S7: OB

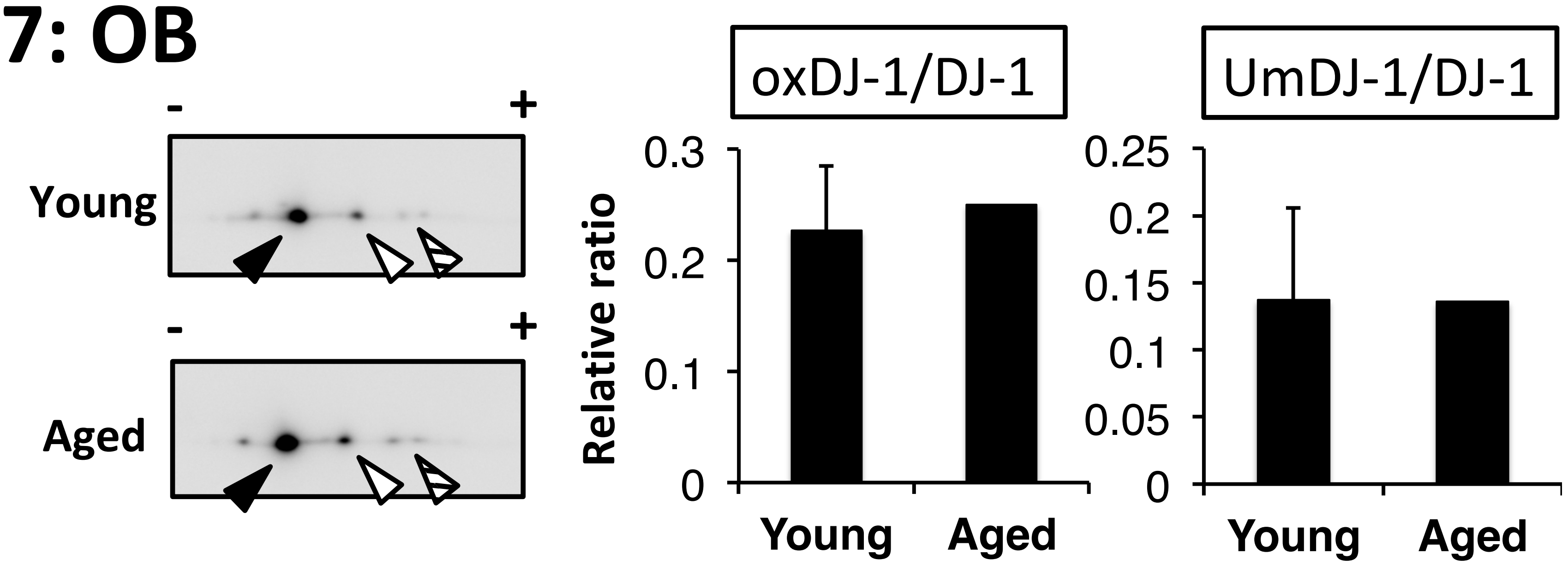


**Supplementary Figure S7 | 2D-PAGE analysis of DJ-1 in the olfactory bulb of aged mice.** Proteins in lysates from the olfactory bulb of young (n = 3) and aged (n = 2) mice were separated by 2D-PAGE and then subjected to western blotting using anti-DJ-1 and anti-oxDJ-1 Ab. Black, white, and striped arrowheads indicate native DJ-1, oxDJ-1, and unknown modified DJ-1 (UmDJ-1). A spot corresponding to oxDJ-1 was only observed in 2D western blot analysis using anti-oxDJ-1 Ab.

# S8: Skeletal muscle

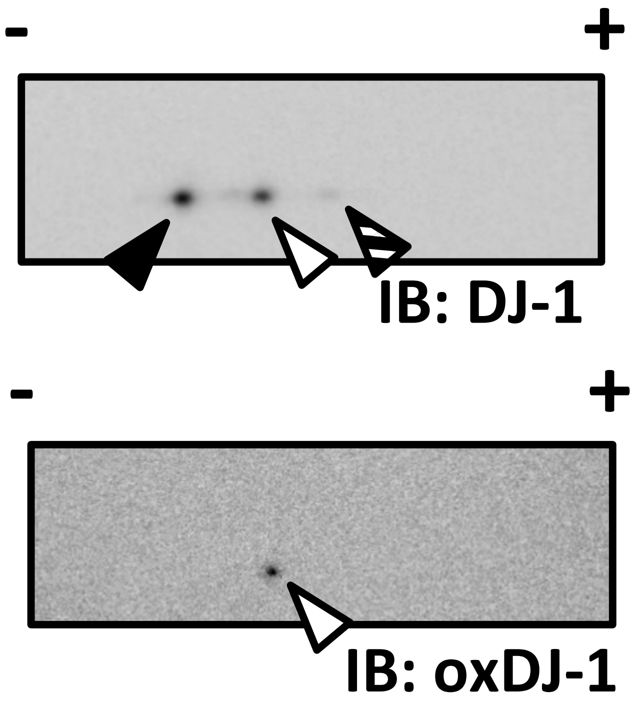


**Supplementary Figure S8 | 2D-PAGE analysis of DJ-1 in the skeletal muscle of aged mice.** Proteins in lysates from skeletal muscle of young mice were separated by 2D-PAGE and then subjected to western blotting using anti-DJ-1 and anti-oxDJ-1 Ab. Black, white, and striped arrowheads indicate native DJ-1, oxDJ-1, and unknown modified DJ-1 (UmDJ-1). A spot corresponding to oxDJ-1 was only observed in 2D western blot analysis using anti-oxDJ-1 Ab.

**S9: SN**

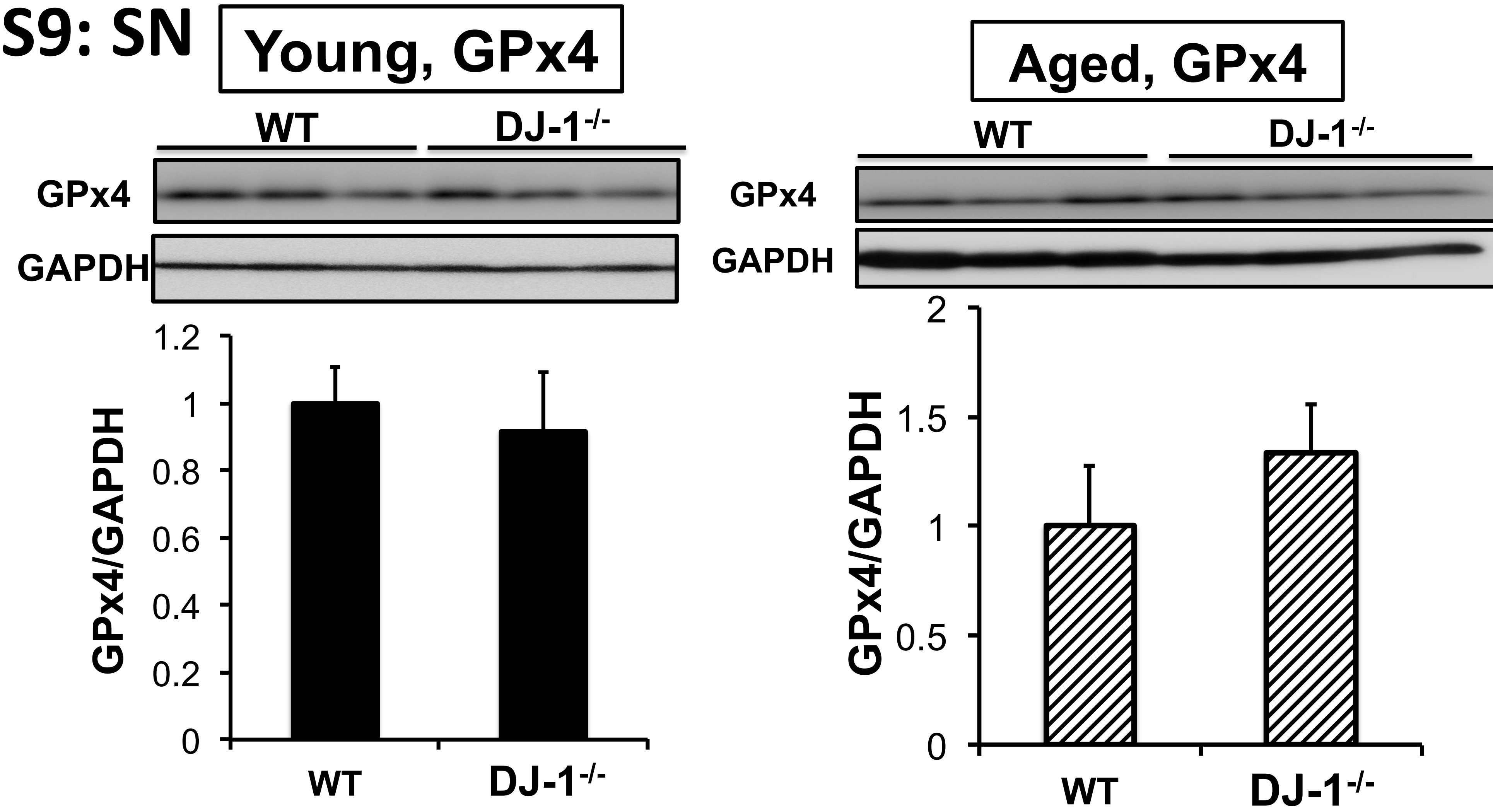


**Supplementary Figure S9 | Levels of glutathione peroxidase 4 (GPx4) in the substantia nigra (SN) of D-1$^{-/-}$ mice.** Young (9 weeks of age, left panel) and aged (more than 100 weeks of age, right panel) mice were analyzed. Protein lysates of the SN of wild-type (WT) and DJ-1$^{-/-}$ mice were subjected to western blot analyses using anti-GPx4 Ab. The relative band densities of GPx4 relative to GAPDH were calculated and are presented as mean ± SD (n = 3-5).

**S10: SN**

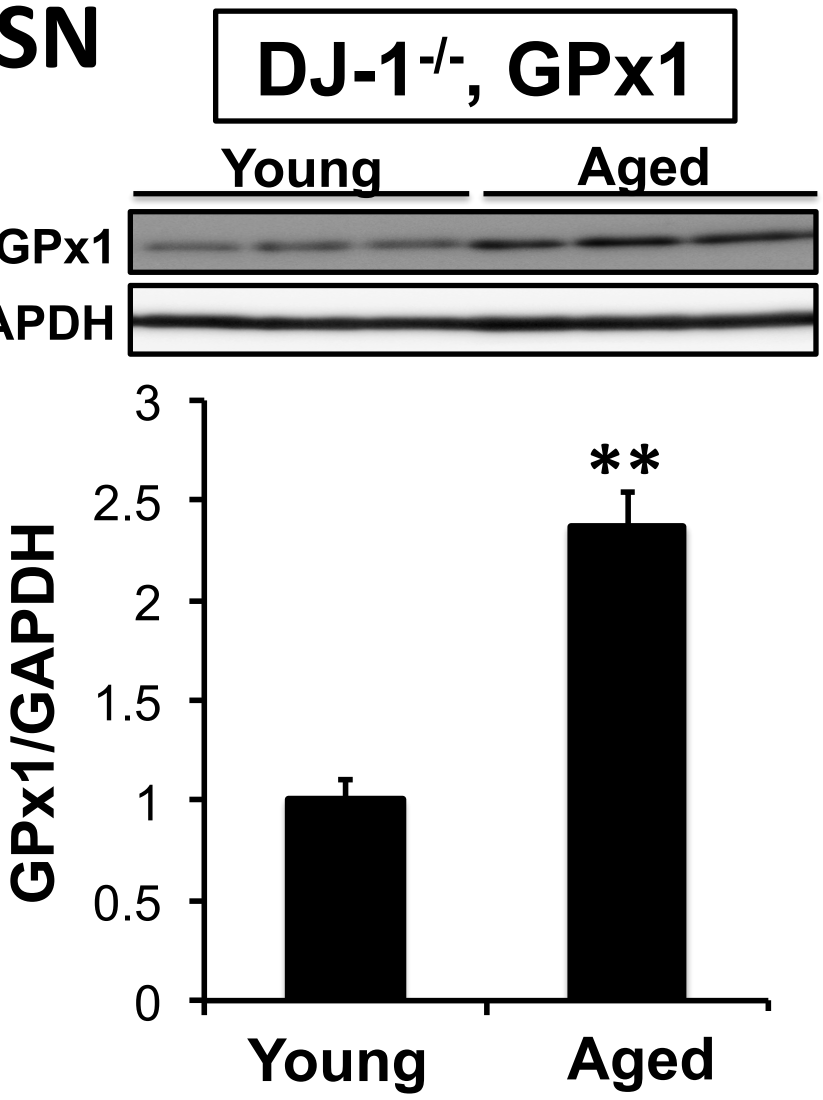


**Supplementary Figure S10 | Elevation of glutathione peroxidase 1 (GPx1) levels in the substantia nigra (SN) of aged D-1$^{-/-}$ mice.** Protein lysates of the SN of young (9 weeks of age, left panel) and aged (more than 100 weeks of age, right panel) DJ-1$^{-/-}$ mice were subjected to western blot analyses using anti-GPx1 Ab. The relative band densities of GPx1 relative to GAPDH were calculated and are presented as mean ± SD (n = 4-5). ** $P$ < 0.01, Student's $t$-test.

# S11: SN

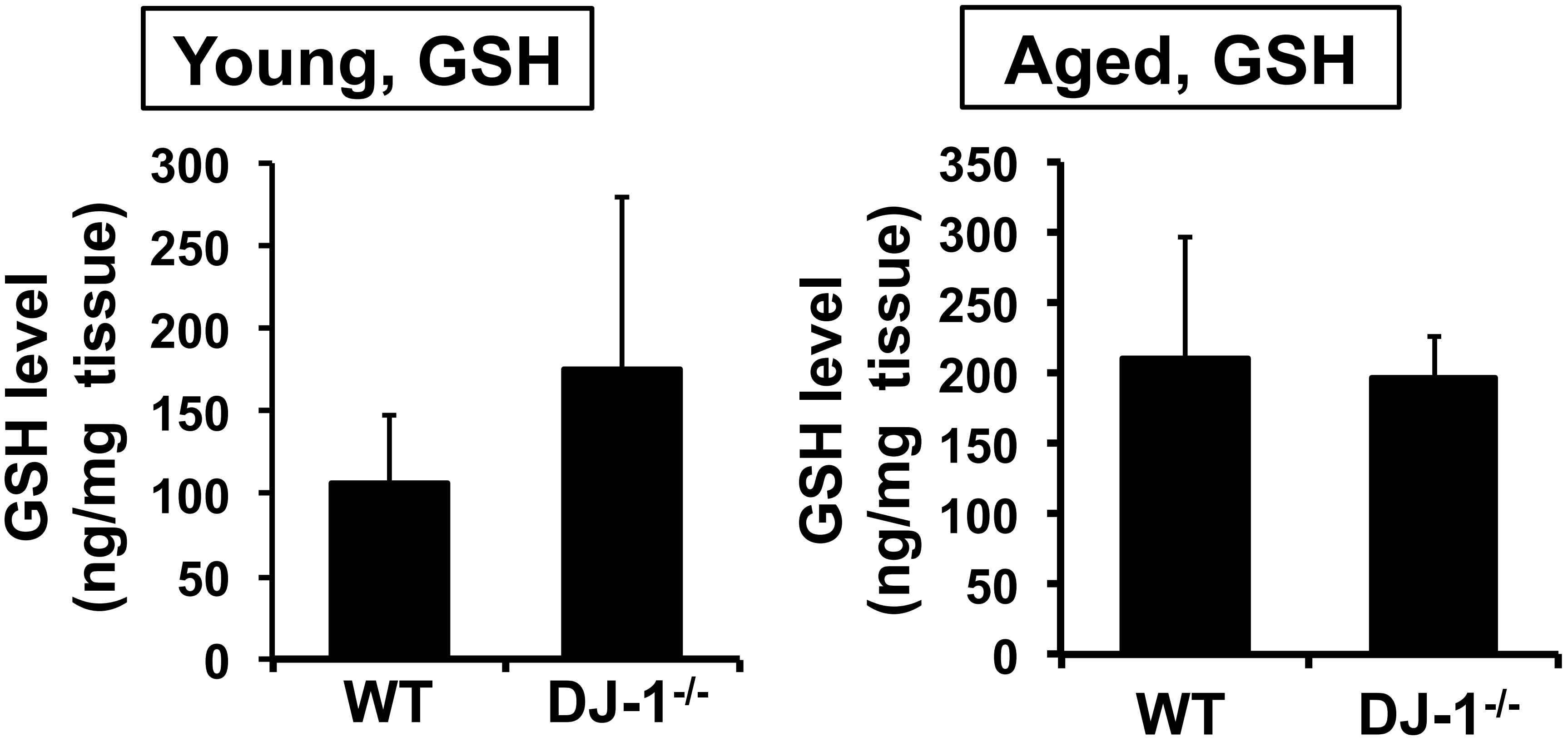


**Supplementary Figure S11 | The levels of reduced form of glutathione (GSH) in the substantia nigra (SN) of D-1$^{-/-}$ mice.** Young (9 weeks of age, left panel) and aged (more than 100 weeks of age, right panel) mice were analyzed. Extracts of the SN of wild-type (WT) and DJ-1$^{-/-}$ mice were subjected to GSH measurements. The determinants, ng GSH per mg tissues, were calculated and are presented as mean ± SD (n = 3-5).

# S12: OB

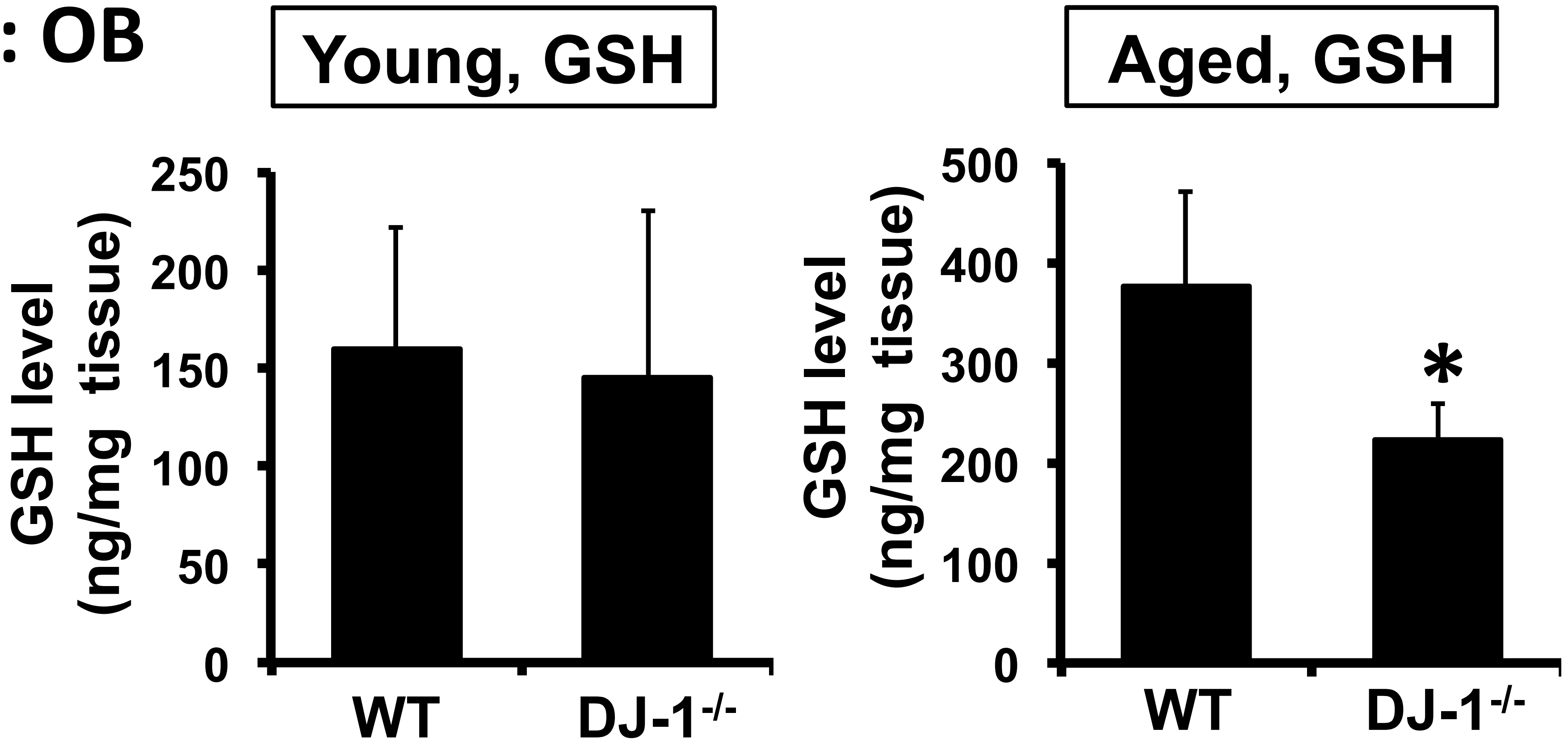


**Supplementary Figure S12 | The levels of reduced form glutathione (GSH) in the olfactory bulb (OB) of D-1$^{-/-}$ mice.** Young (9 weeks of age, left panel) and aged (more than 100 weeks of age, right panel) mice were analyzed. Extracts of the OB of wild-type (WT) and DJ-1$^{-/-}$ mice were subjected to GSH measurements. The determinants, ng GSH per mg tissues, were calculated and are presented as mean ± SD (n = 3-5). * $P < 0.05$, Student's *t*-test.

# S13: OB

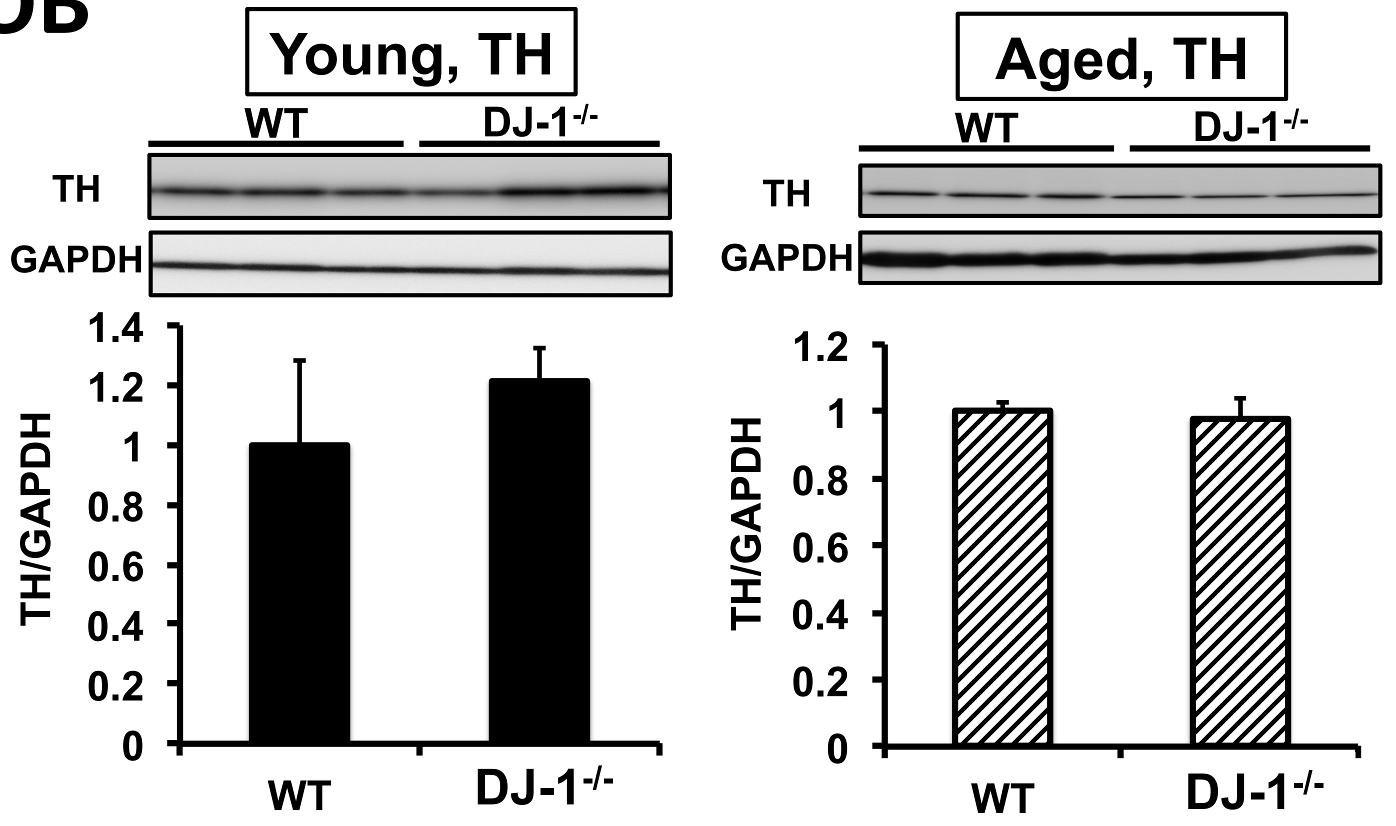


**Supplementary Figure S13 | The levels of tyrosine hydroxylase (TH) in the olfactory bulb (OB) of D-1$^{-/-}$ mice.** Young (9 weeks of age, left panel) and aged (more than 100 weeks of age, right panel) mice were analyzed. Extracts of the OB of wild-type (WT) and DJ-1$^{-/-}$ mice were subjected to western blot analyses using anti-TH Ab. The relative band densities of TH relative to GAPDH were calculated and are presented as mean ± SD (n = 3-5).

# S14: OB

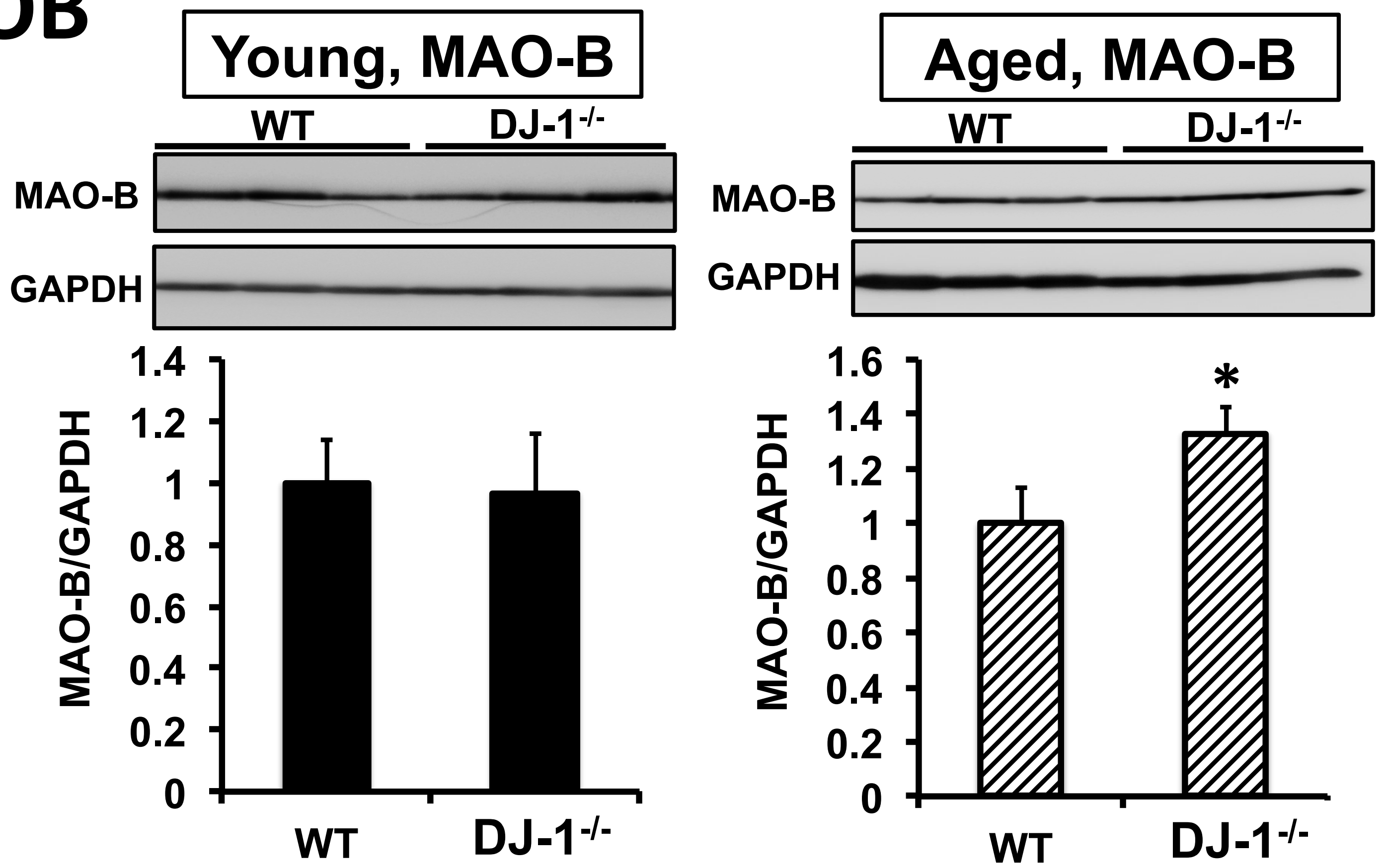


**Supplementary Figure S14 | The levels of monoamine oxidase type B (MAO-B) in the olfactory bulb (OB) of D-1$^{-/-}$ mice.** Young (9 weeks of age, left panel) and aged (more than 100 weeks of age, right panel) mice were analyzed. Extracts of the OB of wild-type (WT) and DJ-1$^{-/-}$ mice were subjected to western blot analyses using anti-MAO-B Ab. The relative band densities of MAO-B relative to GAPDH were calculated and are presented as mean ± SD (n = 3-5).

# S15: OB

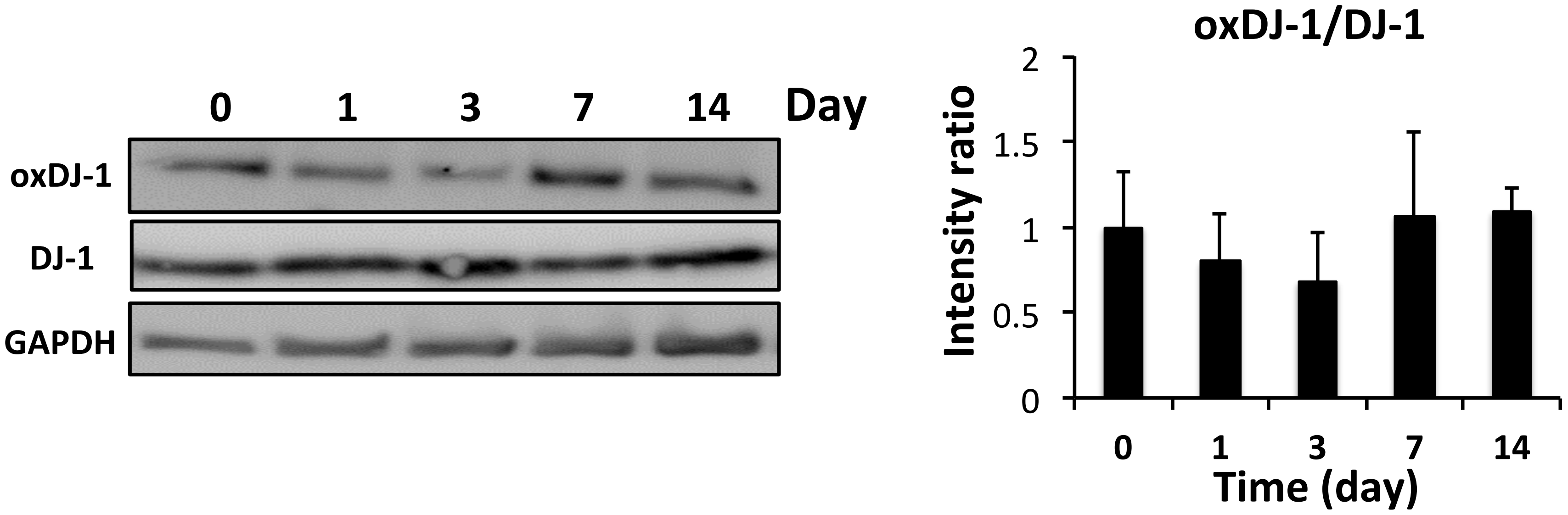


**Supplementary Figure S15 | The change of oxDJ-1 levels in the olfactory bulb (OB) of MPTP-treated mice.** After the administration of PBS and MPTP (15 mg/kg, 3 times i.p.), protein lysates of the OB at indicated time were subjected to western blot analyses using each specific Ab. The relative band densities of oxDJ-1 relative to DJ-1 were calculated and are presented as mean ± SD (n = 4).

# S16: Ctx

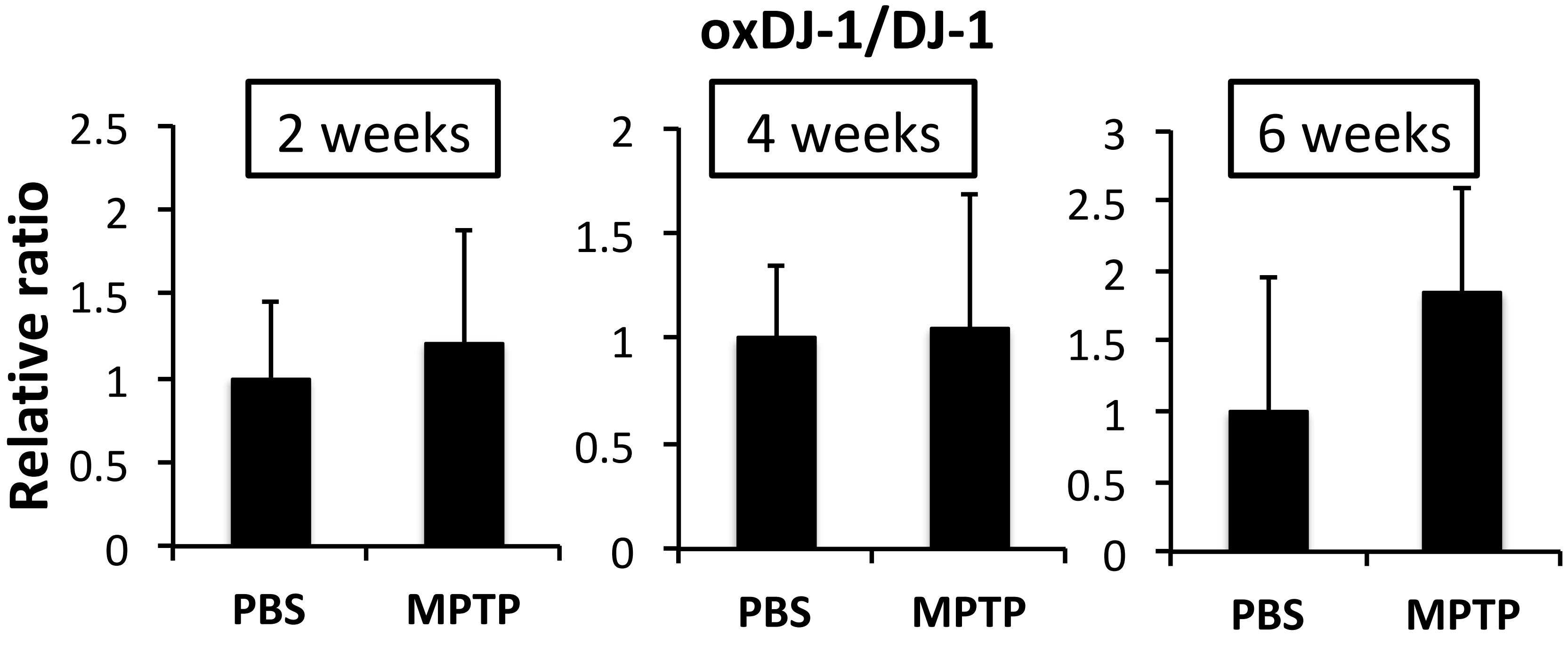


**Supplementary Figure S16 | Levels of oxDJ-1 in the cortex (Crx) of MPTP-treated mice.** After the administration of PBS and MPTP (15 mg/kg, 3 times i.p.), protein lysates of the Ctx at indicated time were subjected to western blot analyses using each specific Ab. The relative band densities of oxDJ-1 relative to DJ-1 were calculated and are presented as mean ± SD (n = 6).

# S17: Cb

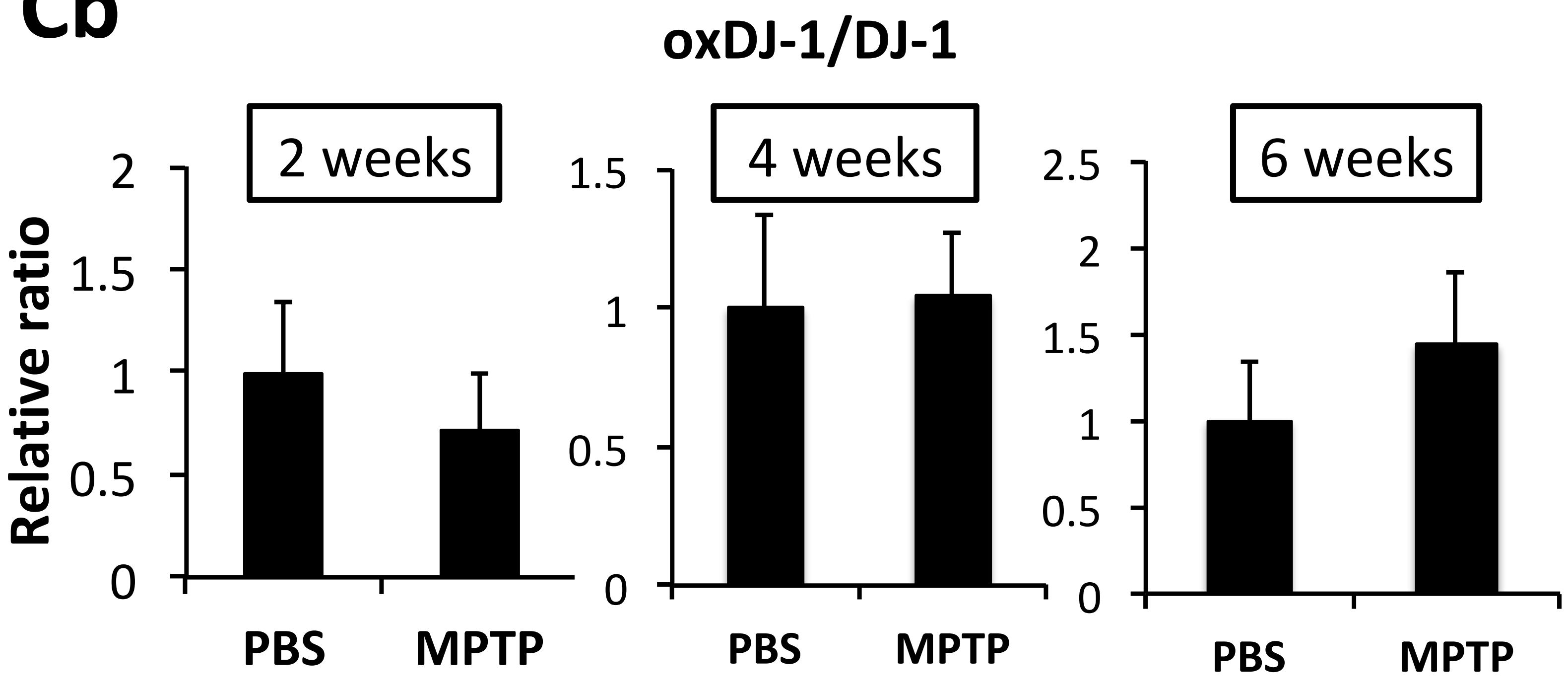


**Supplementary Figure S17 | Levels of oxDJ-1 in the cerebellum (Cb) of MPTP-treated mice.** After the administration of PBS and MPTP (15 mg/kg, 3 times i.p.), protein lysates of the Cb at indicated time were subjected to western blot analyses using each specific Ab. The relative band densities of oxDJ-1 relative to DJ-1 were calculated and are presented as mean ± SD (n = 6).

# S18: Hp

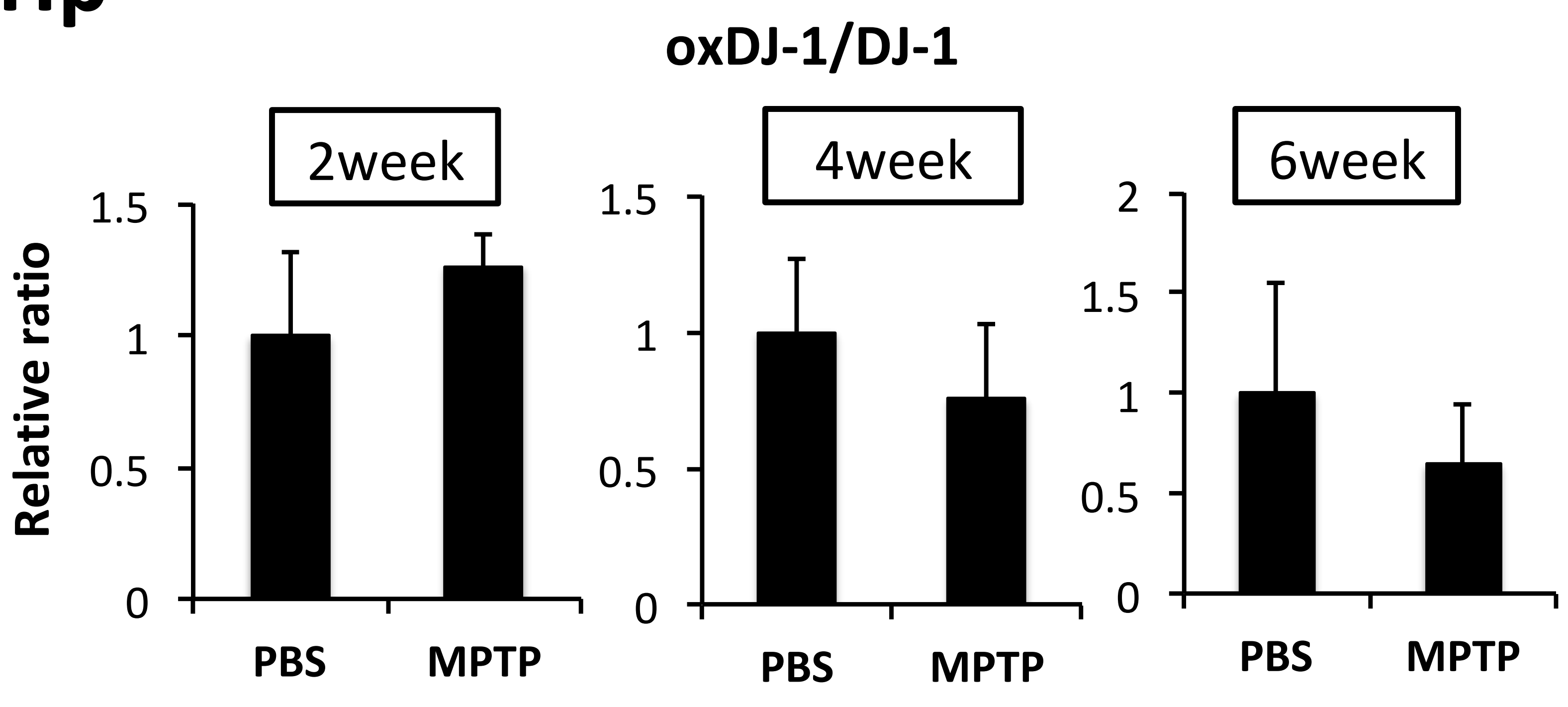


**Supplementary Figure S18 | Levels of oxDJ-1 in the hippocampus (Hp) of MPTP-treated mice.** After the administration of PBS and MPTP (15 mg/kg, 3 times i.p.), protein lysates of the Hp at indicated time were subjected to western blot analyses using each specific Ab. The relative band densities of oxDJ-1 relative to DJ-1 were calculated and are presented as mean ± SD (n = 6).

# S19: Skeletal muscle

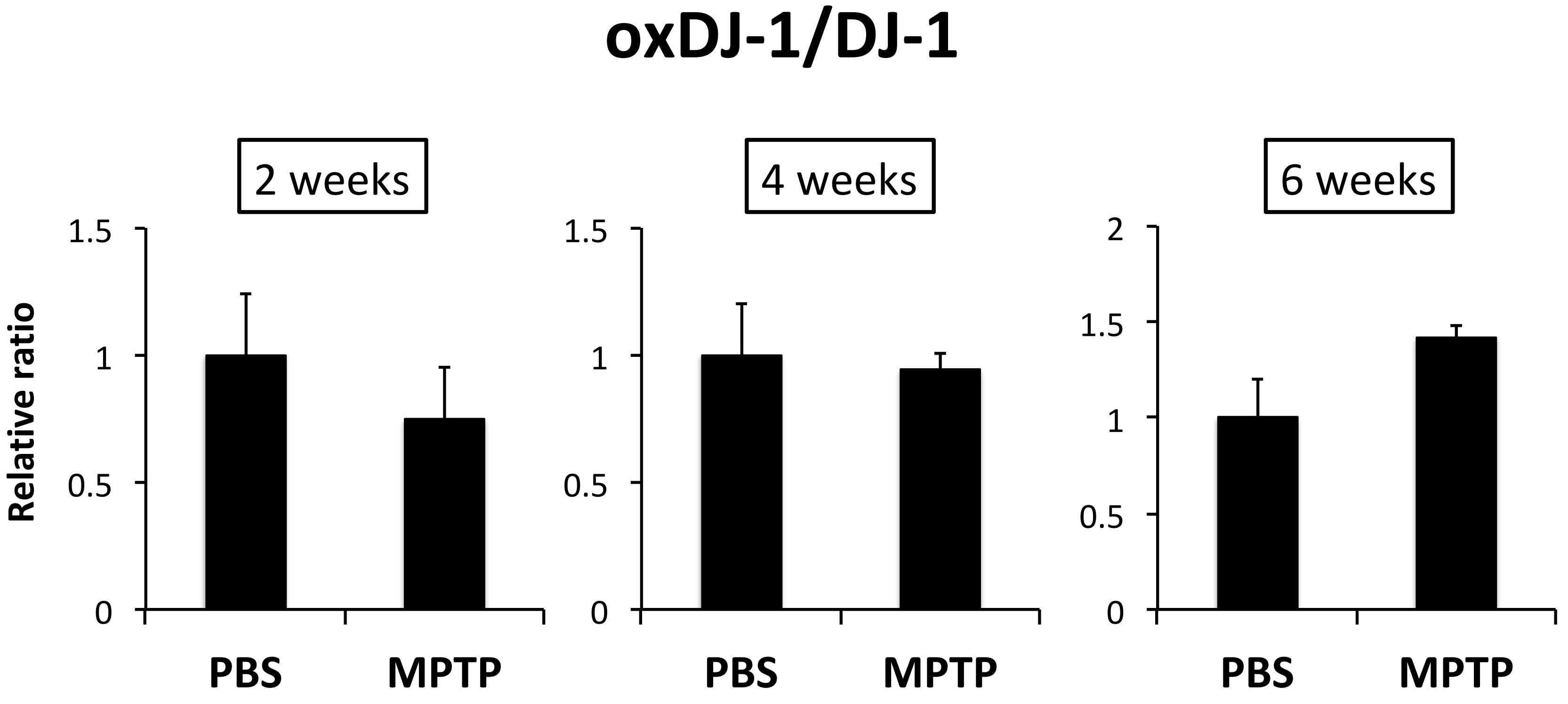


**Supplementary Figure S19 | Levels of oxDJ-1 in the skeletal muscle of MPTP-treated mice.** After the administration of PBS and MPTP (15 mg/kg, 3 times i.p.), protein lysates of the skeletal muscle at indicated time were subjected to western blot analyses using each specific Ab. The relative band densities of oxDJ-1 relative to DJ-1 were calculated and are presented as mean ± SD (n = 6).

# S20

**Fig. 1A, oxDJ-1 blot, right panel**

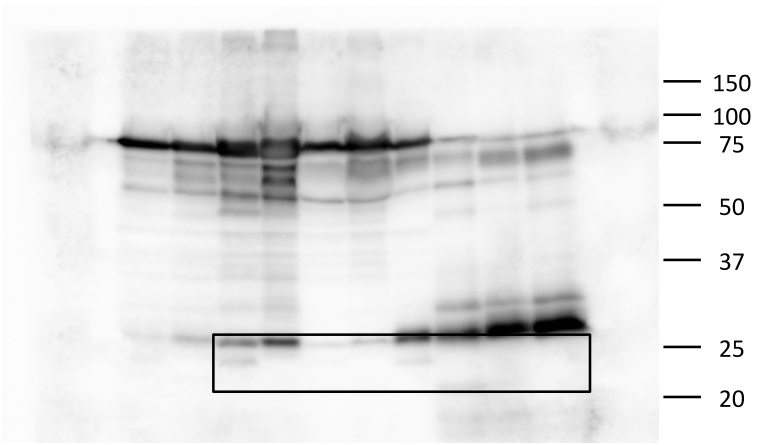


**Fig. 1A, oxDJ-1 blot, left panel**

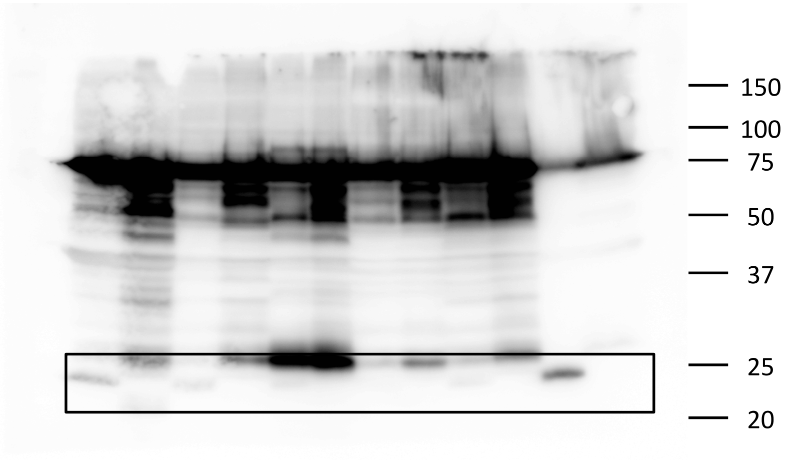


**Fig. 1A, DJ-1 blot, left panel**

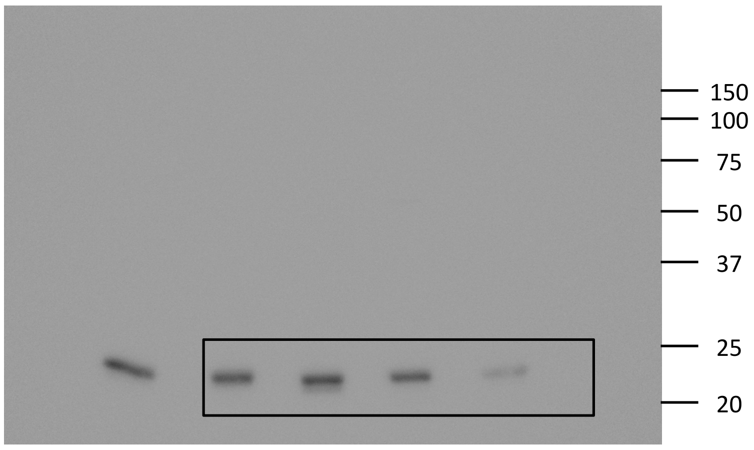


**Fig. 1A, DJ-1 blot, right panel**

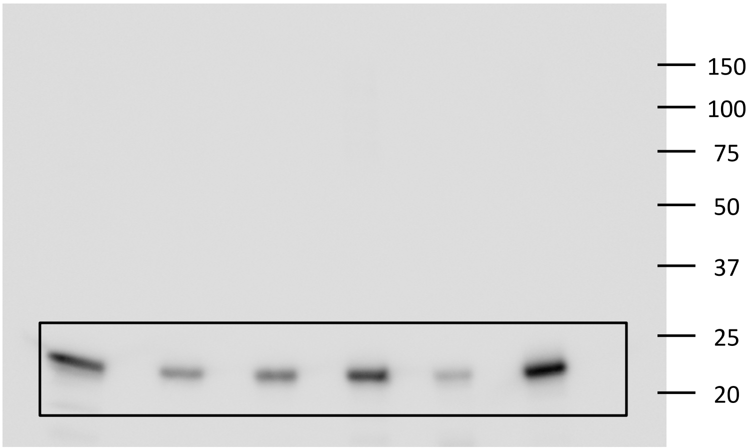


**Fig. 1E, oxDJ-1 blot**

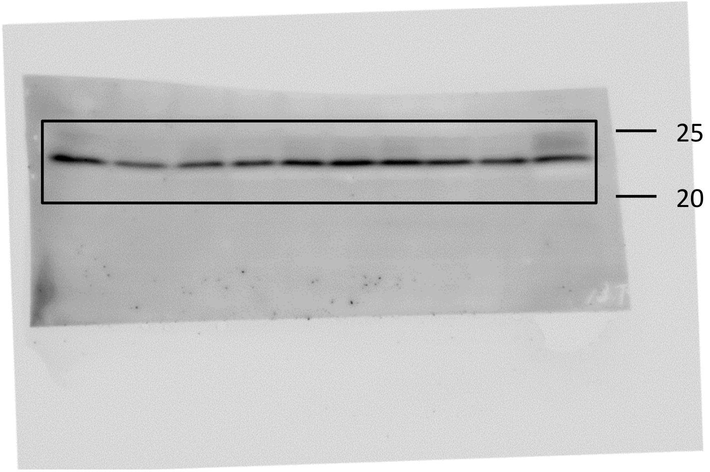


**Fig. 1E, DJ-1 blot**

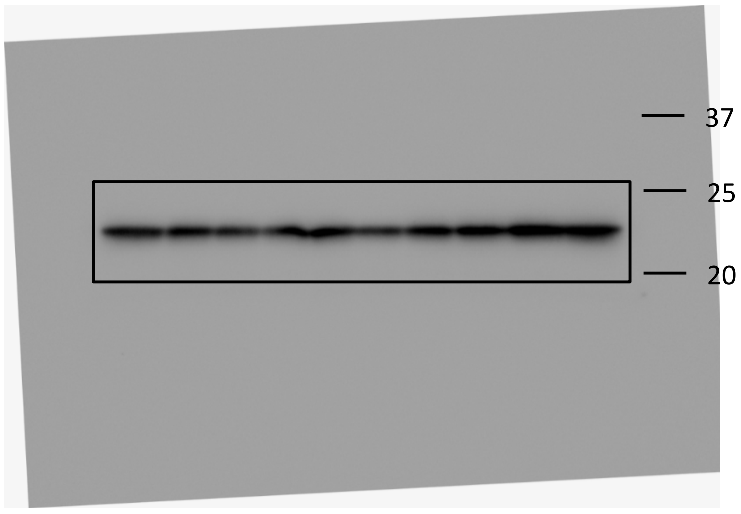


**Supplementary Figure S20 | The full blot corresponding to main figures as indicated.** Used area is enclosed.

## Supplementary Fig. S20, Y. Mita, et al.

# S21

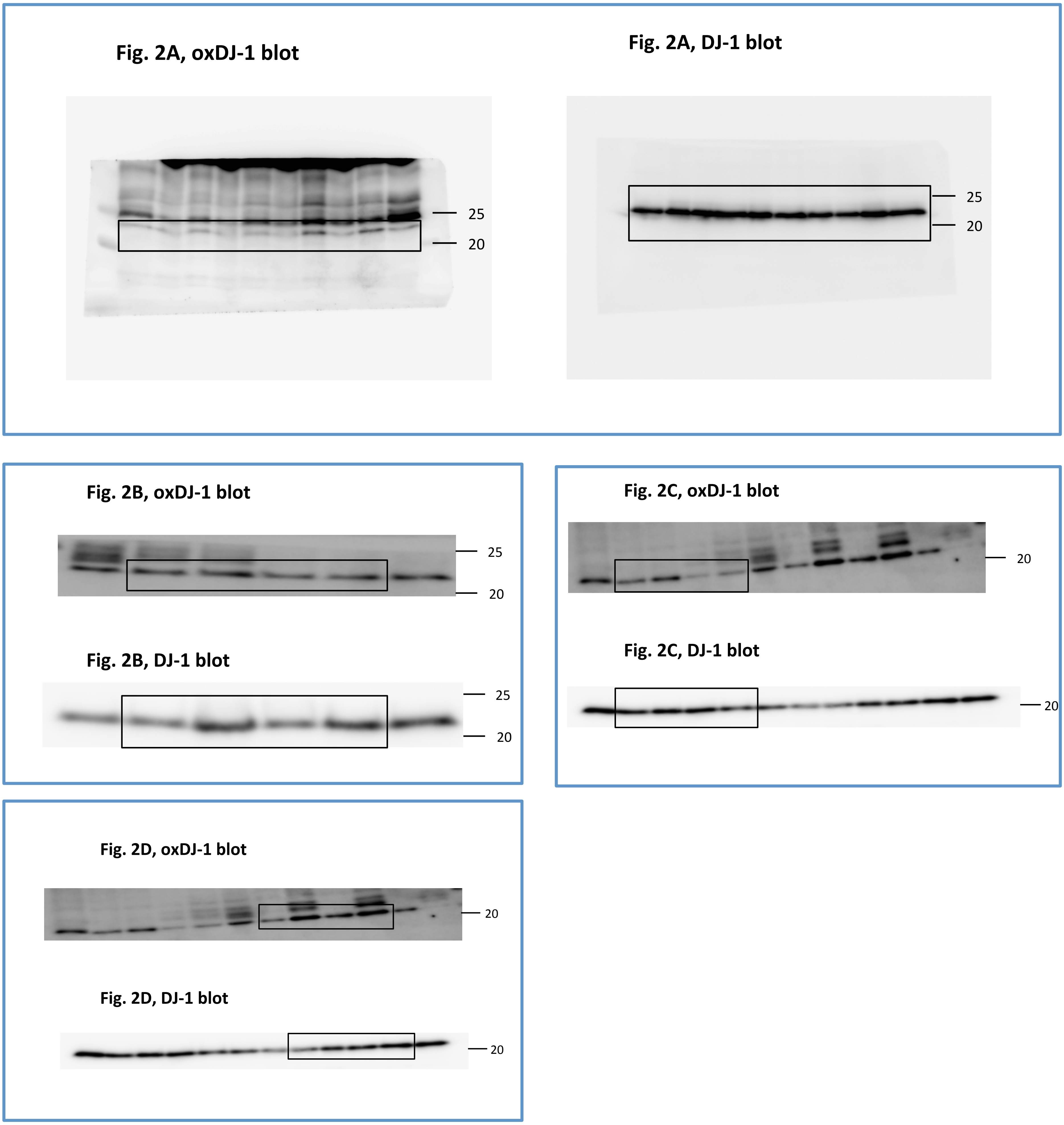


**Supplementary Figure S21 | The full blot corresponding to main figures as indicated.** Used area is enclosed.

## Supplementary Fig. S21, Y. Mita, et al.

# S22

Fig. 3A, oxDJ-1 blot, Young

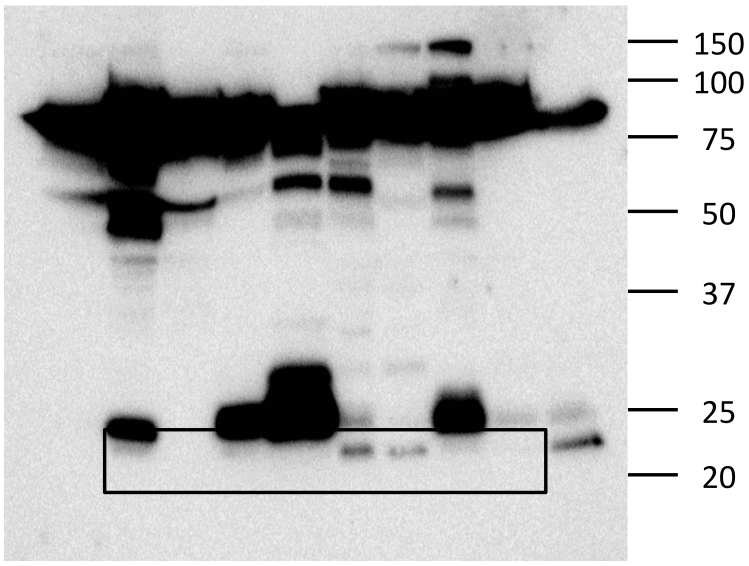


Fig. 3A, oxDJ-1 blot, Aged

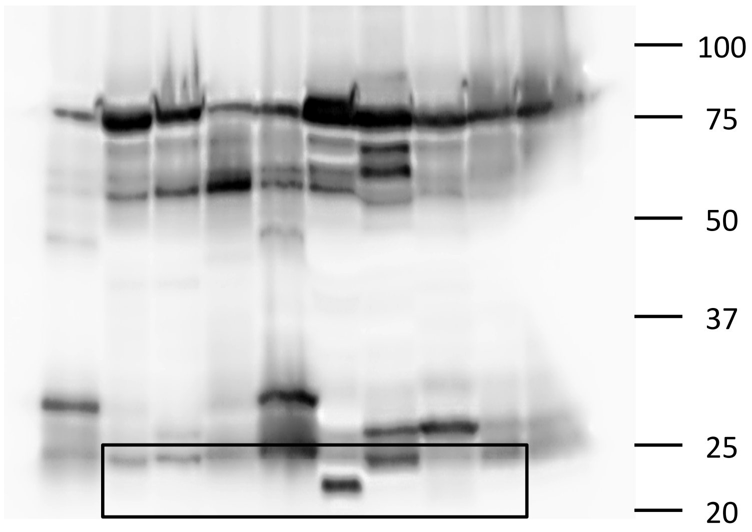


Fig. 3A, DJ-1 blot, Young

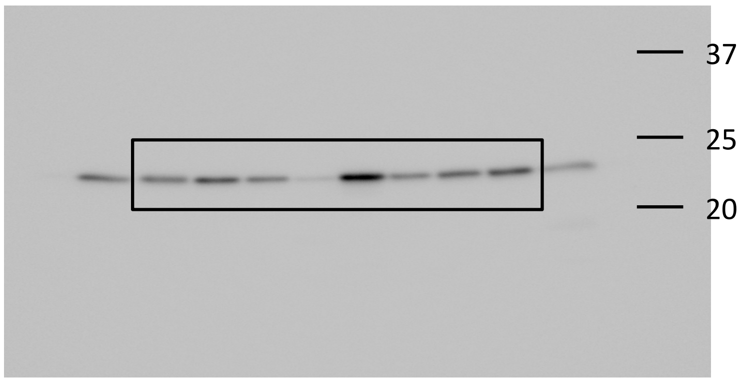


Fig. 3A, DJ-1 blot, Aged

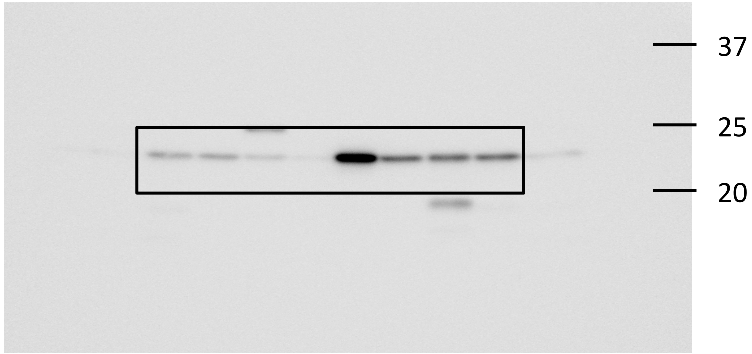


Fig. 3B, oxDJ-1 blot, Heart (Left)
Fig. 3D, oxDJ-1 blot, Skeletal muscle (Right)

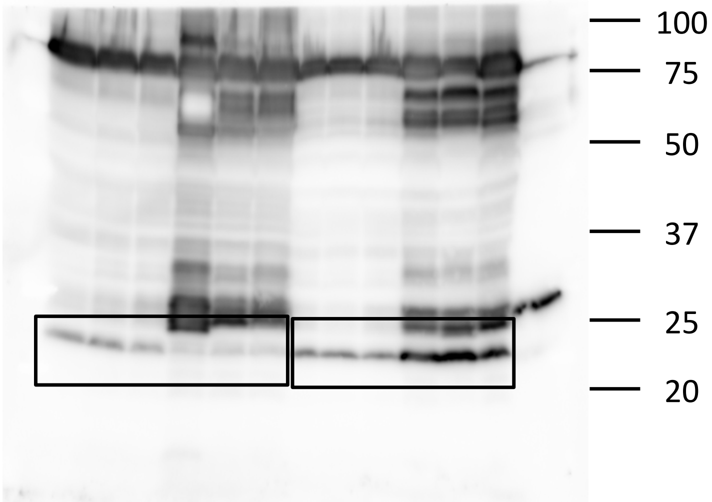


Fig. 3B, DJ-1 blot, Heart (Left)
Fig. 3D, DJ-1 blot, Skeletal muscle (Right)

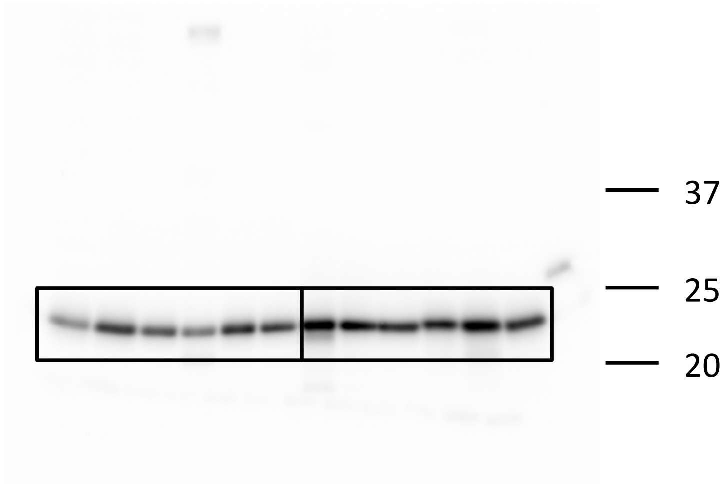


**Supplementary Figure S22 | The full blot corresponding to main figures as indicated.** Used area is enclosed.

Supplementary Fig. S22, Y. Mita, et al.
